# Direct‐Ink‐Writing Printed Stretchable Eutectic Gallium–Indium Antenna for Robust Wireless Communication

**DOI:** 10.1002/advs.202414285

**Published:** 2025-04-02

**Authors:** Xiangyu Guo, Yufei Liu, Zhizhou Zhou, Haotian Peng, Yerun Gao, Ming Shao, Yu Yu

**Affiliations:** ^1^ Wuhan National Laboratory for Optoelectronics and School of Optical and Electronic Information Huazhong University of Science and Technology Wuhan 430074 China; ^2^ Optics Valley Laboratory Hubei 430074 China

**Keywords:** direct‐ink printing, stretchable antenna, liquid metal, flexible electronics

## Abstract

Stretchable antennas represent a pivotal innovation in enhancing wireless interconnection and driving the proliferation of Internet of Things (IoT) applications. Eutectic Gallium–Indium (EGaIn) is an ideal conductor for stretchable antennas. However, the inherent high surface tension and fluidity of EGaIn make the patterning low‐precision, time‐consuming, and failure‐prone. Here a wideband stretchable antenna is presented by direct writing of thermoplastic polyurethane‐modified EGaIn ink and activated by water‐bath ultrasound. The ink exhibits printing‐friendly rheological properties and surface energy, enabling high patterning‐precision printing (10 µm) while preserving excellent conductivity (1.6 × 10^6^ S m^−1^). Benefiting from these improvements, the printed antenna achieves a large fractional bandwidth (75%), a high radiation efficiency (76.6%), and an exceptional ultimate strain (> 240%). For a proof‐of‐concept demonstration, the antenna enables a 50‐meter wireless communication, under the case of 240% stretching or conformally wrapped around a drone. This work provides an efficient and universal strategy for manufacturing stretchable antennas, with broad potential in advanced IoTs technologies.

## Graphical abstract

In summary, a universal EGaIn‐based DIW printing strategy is presented for fabricating wideband stretchable antennas. The printed antenna achieves an operating bandwidth of 3.75–8.21 GHz (fractional bandwidth: 75%), a peak gain of 1.8 dB and a radiation efficiency of 76.6%, surpassing state‐of‐the‐art stretchable antennas. More importantly, the antenna maintains stable wireless communication even under 240% strain and conformal wrapping.

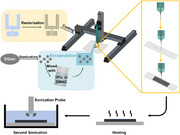

## 1 Introduction

As the converters between guided waves and radiated electromagnetic waves, antennas have been regarded as indispensable components in wireless systems for decades. Recently, there have been emerging commercial applications for wearable electronics and soft robotics in Internet of Things (IoT) scenarios.^[^ ^1^, ^2^, ^3^, ^4^, ^5^ ^]^ These stretchable electronics require stretchable wireless interfaces for information interaction. Therefore, it is important to develop stretchable antennas to realize efficient wireless links between stretchable electronics and external systems for real‐time communication and control.

Stretchable antennas require both high conductivity (>10^6^ S m^−1^) and stretchability (> 30%). Although highly conductive metals including copper and aluminum are ideal for traditional antennas, they are rigid and can hardly be stretched. Therefore, intrinsically stretchable conductive materials are proposed for stretchable antennas, but the trade‐off between stretchability and conductivity remains. For instance, typical polymer conductor poly (3,4‐ethylenedioxythiophene): poly (styrene sulfonate) (PEDOT: PSS) exhibits excellent stretchability but suffers from low conductivity (<1 × 10^5^ S m^−1^).^[^ ^6^, ^7^ ^]^ On the other hand, the nanomaterials (graphene, MXene, Ag nanowires) and their nanocomposites demonstrate higher conductivity (≈10^6^ S m^−1^), but the stretchability is lower than 20% and exhibit strain‐variant resistance.^[^ ^8^, ^9^, ^10^, ^11^, ^12^, ^13^ ^]^ Consequently, further applications of these materials in stretchable antennas remain elusive.

In contrast, Eutectic Gallium–Indium (EGaIn) stands out as a competitive candidate because of its high conductivity (3.4 × 10^6^ S m^−1^), extreme mechanical stretchability, and biocompatibility.^[^ ^14^, ^15^, ^16^ ^]^ Recently, EGaIn has been patterned for various stretchable electronic devices,^[^ ^17^, ^18^ ^]^ and one typical patterning method is injecting EGaIn into hermetic microfluidic channels.^[^ ^19^, ^20^, ^21^ ^]^ Unfortunately, these channels must be interconnected, making it difficult to create dense or isolated patterns.^[^ ^22^ ^]^ Besides, the injection requires an inlet port, leading to unexpected leakage under strain.^[^ ^21^, ^23^ ^]^ Another promising method involves directly printing EGaIn to specific patterns utilizing Direct‐Ink‐Writing (DIW).^[^ ^24^, ^25^ ^]^ DIW is an extrusion‐based patterning technique that can fabricate customized high‐resolution 3D structures by manipulating ink extrusion and stacking layer by layer. This technique can print preset patterns directly on almost any substrate, whether rigid or stretchable, without time‐consuming masks and transfer processes.^[^ ^26^, ^27^ ^]^ However, the low viscosity of EGaIn results in high flowability and easy collapse, impeding the formation of well‐defined printed patterns.^[^ ^22^ ^]^ Moreover, the high surface tension hinders EGaIn from wetting the substrate effectively.^[^ ^28^, ^29^, ^30^ ^]^ As a result, EGaIn can only be printed as isolated lines at specific widths rather than being stacked or interconnected into layers, which restricts the design flexibility of antennas.^[^ ^24^, ^31^ ^]^ To facilitate the printing process, recent studies have developed various composites to modify the surface properties of EGaIn. However, these approaches rely on breaking the oxide layer on the EGaIn surface (laser sintering and tensile stress, etc.) to activate conductive networks.^[^ ^32^, ^33^, ^34^ ^]^ These treatments inevitably cause the leakage of EGaIn (especially under stretching), resulting in environmental pollution and device failure.^[^ ^35^ ^]^ Hence, it is highly significant to explore a more universal strategy for EGaIn patterning and apply it to manufacture antennas.

Here, we develop a strategy for patterning EGaIn using DIW printing and apply it to fabricate a stretchable antenna. By introducing thermoplastic polyurethane (TPU) as the modifier, the EGaIn‐TPU composite (ETC) ink exhibits improved surface energy and rheological properties for printing. This improvement facilitates successive ink stacking and improves pattern fidelity. The printed patterns demonstrate leakage‐free stretchability and high conductivity, well‐suited for manufacturing stretchable antennas with high radiation efficiency. Leveraging these advantages, we present a wideband stretchable antenna with an exceptional ultimate strain of >240%. The measured operating bandwidth of the antenna ranges from 3.75 to 8.21 GHz (a fractional bandwidth of 75%), with a radiation efficiency of 76.6%. Furthermore, 50‐meter wireless communication experiments of the antenna are achieved upon both stretched and conformally bent states.

## 2 Result

### 2.1 The DIW Printing System and Ink Formulation

**Figure 1:**
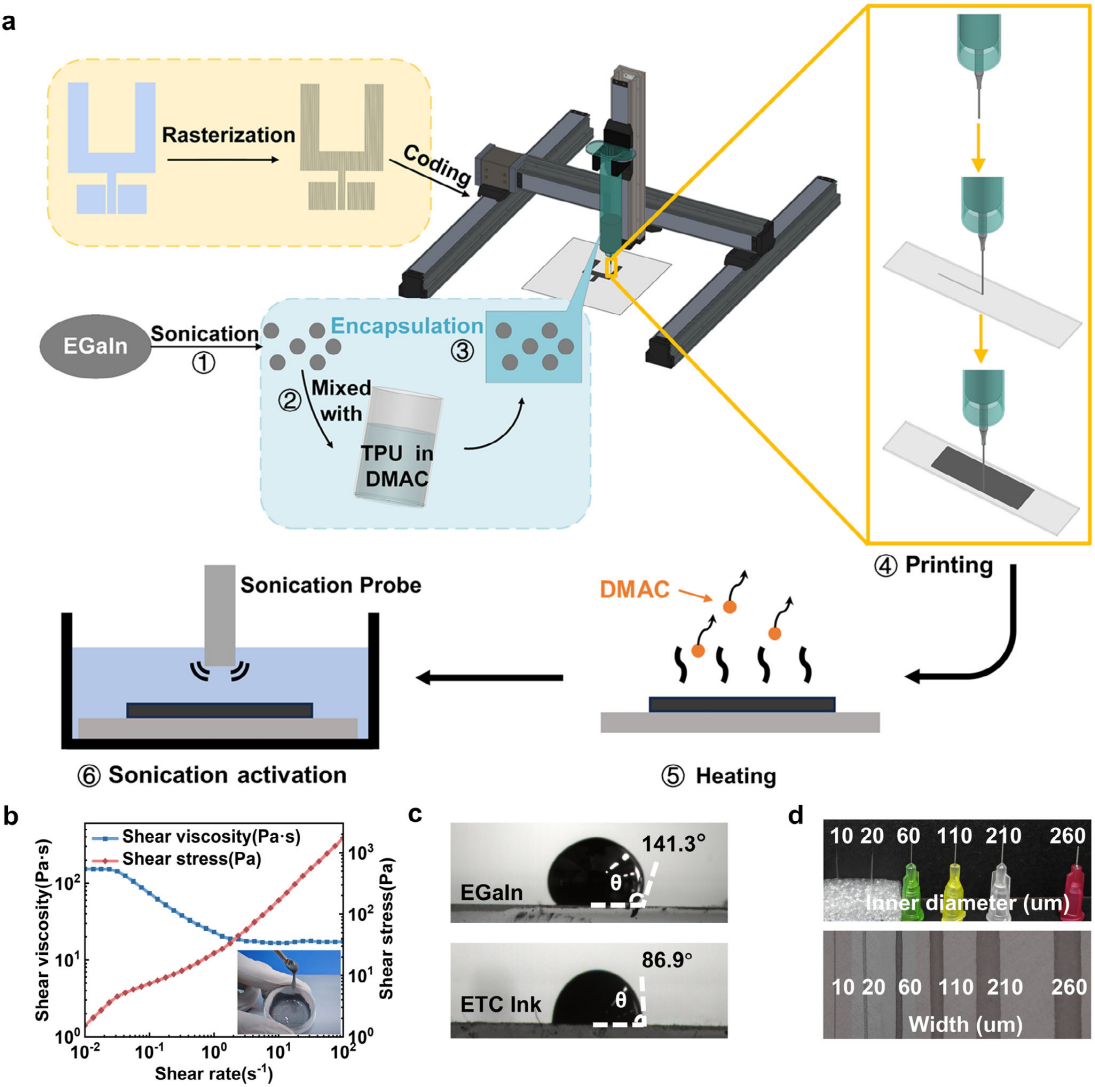
The schematic diagram of the DIW printing system and rheological properties of the ETC ink. a) The schematic diagram of the DIW printing process and the formulation of the ETC ink. b) Shear stress and shear viscosity of the ETC ink at different shear rates. The inset is the ETC ink under test. c) The contact angles between the TPU substrate and the pristine EGaIn/the ETC ink. d) The mono‐fiber printing experiment using needles with various diameters.

**Figure** **1**a illustrates the process of patterning EGaIn utilizing the DIW printing system (See Experimental Section for more details). The DIW system mainly consists of a nozzle connected to an ink reservoir, a precise three‐axis motion platform, and a pneumatic liquid dispenser. The printing process involves two critical steps: extruding the ink from the dispenser to form the lines, and stacking lines to a layer. In the DIW process of EGaIn, the extrusion of EGaIn lines relies on the confinement caused by the dense oxide layers that rapidly form in ambient air as a ‘shell’.^[^ ^24^, ^31^ ^]^ However, a significant challenge arises when printed lines are stacked into a layer. To ensure the formation of continuous 2D conductive patterns, adjacent lines are printed with a spacing *ls* smaller than the line width *l* (*ls* < *l*. Figure S1, Supporting Information).^[^ ^36^ ^]^ And challenges arise when printing new lines that partially overlap existing ones. The newly extruded EGaIn tends to infiltrate into the ‘oxide shell’ of the previously printed lines, causing expansion of the shell and weakening its structural integrity. This results in the 3D stacking of EGaIn lines being deemed incompatible. Therefore, to successfully achieve large‐area pattern printing, the inks should possess specific characteristics: appropriate shear‐thinning behavior for smooth extrusion, an appropriate contact angle for stable adhesion to the substrate, and high viscosity to prevent structural collapse after extrusion.

Therefore, EGaIn is modified as a composite ink for a simple and universal DIW system. EGaIn is broken into microparticles by sonication and then mixed with TPU solution in dimethylacetamide (DMAC) to form the ETC ink. As shown in Figure 1b, compared to pristine EGaIn with a low viscosity of 2 × 10^−3^ Pa s, the formulated ETC ink (the inset of Figure 1b) exhibits high viscosity (153 Pa s) which decreases with increasing shear rates. The ink presents a clear shear‐thinning behavior and non‐Newtonian fluid characteristics for the continuous extrusion printing process.^[^ ^37^ ^]^ Furthermore, the high surface energy of DMAC efficiently enhances the overall surface energy of the ETC ink. Consequently, the pristine EGaIn exhibits a contact angle of 141.3° on the TPU substrate while the ETC ink has a reduced angle of 86.9° (Figure 1c). These changes lead to the excellent printability and high shape fidelity of the ETC ink.^[^ ^38^ ^]^ Considering the viscoelastic curves and printing parameter experiment (Figures S1 and S2, Supporting Information), the print speed is set as 0.8 mm s^−1^ with an air pressure of 660 kPa. As shown in Figure 1d, the ETC ink is validated to be extruded through needles with various diameters from 10 to 160 µm, all of which provide continuous mono‐fiber printing. Notably, the fluidity of EGaIn allows for clog‐free and stable extrusion through a 10 µm nozzle (Figure S3 and Video S1, Supporting Information), overcoming the conventional 10–20× nozzle‐to‐particle size ratio limitation required for solid‐particle‐based inks.^[^ ^39^ ^]^ This capability extends the applicability of our method to higher‐resolution printing, which is essential for the fabrication of high‐frequency radio frequency (RF) devices that typically require smaller feature sizes.^[^ ^40^ ^]^

### 2.2 The Electrical and Mechanical Characterization of the Printed ETC

**Figure 2:**
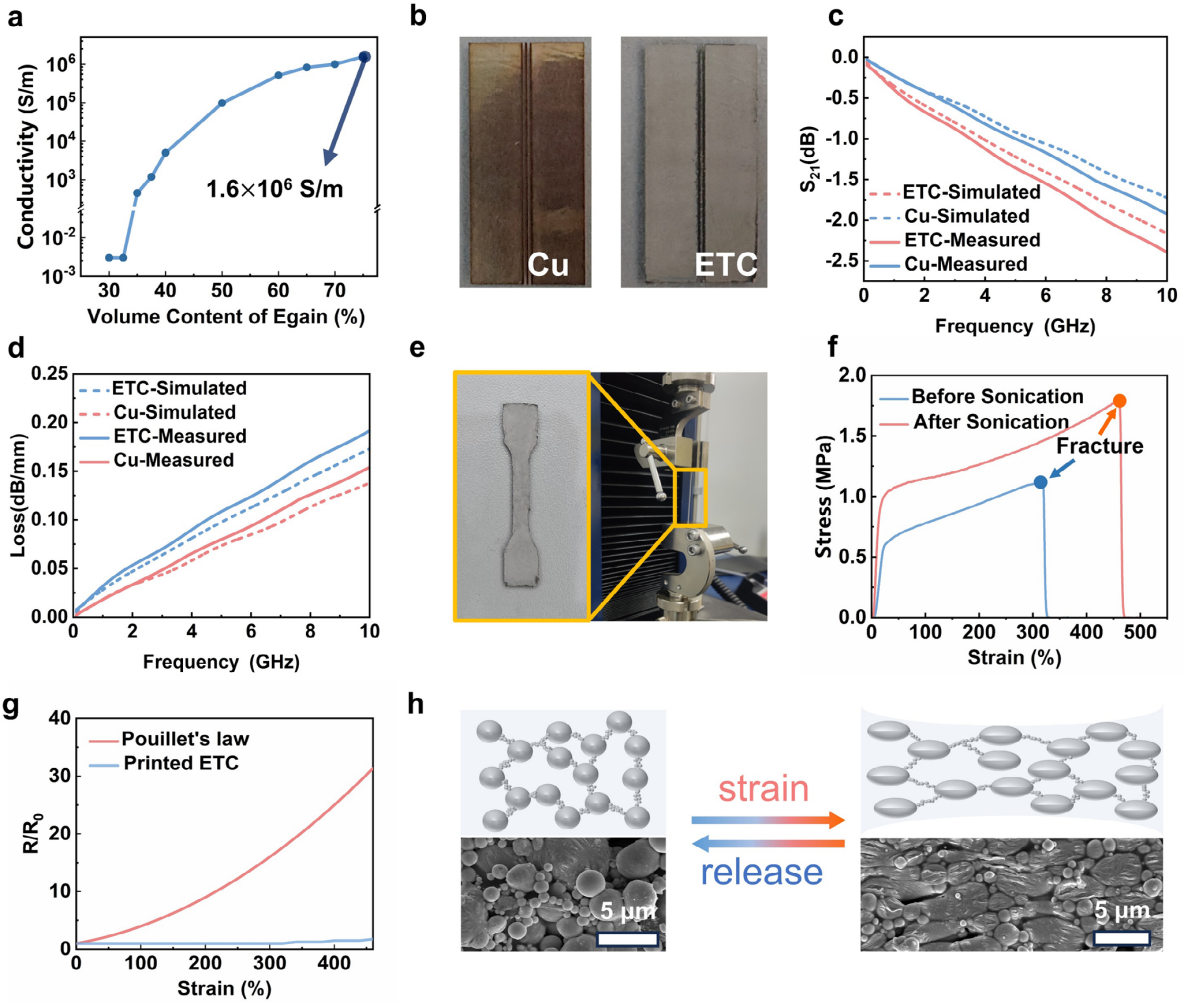
The electrical and mechanical characterization of the ETC. a) The conductivity of the ETC as the EGaIn content increases from 30 to 75 vol%. b) The photograph of the CPW transmission line. c) The S_21_ of the CPW transmission line and the Cu transmission line, respectively. d) The transmission loss of the printed ETC transmission line and the Cu transmission line, respectively. e) The ETC printed on glass and patterned into a “dog‐bone” for the tensile experiment. f) The stress–strain curves of the ETC before and after sonication. g) The relative resistance‐strain curves of the printed ETC and Pouillet's law. h) The schematic diagrams and SEM images of the sonicated composite upon 60% strain.

As shown in Figure 1a, the printed ETC pattern (initially in a liquid state containing DMAC solvent) solidifies upon solvent removal through heating at 60 °C for 4 h. A subsequent sonication process then activates the solidified ETC pattern, transitioning it from an insulative to a conductive state. The conductivity changes before and after activation are measured using the four‐probe method (see the Experimental Section for details). The measured conductivity of the ETC pattern increases from 3 × 10^−3^ to 1.6 × 10^6^ S m^−1^ after the second sonication process, which is on the same order of magnitude as that of the pristine EGaIn (3 × 10^6^ S m^−1^). This enhancement is attributed to the formation of extra conductive pathways, as observed at the microscopic level through scanning electron microscopy (SEM) images (Figure S4, Supporting Information). Besides, the conductivity of the ETC is directly related to the EGaIn volume fraction. As shown in **Figure** **2**a, the conductivity rapidly increases from 450 to 1.6 × 10^6^ S m^−1^ as the content of EGaIn increases from 35% to 75% by volume. The high conductivity of the printed ETC contributes to low RF loss. To validate this, copper and ETC coplanar waveguide (CPW) transmission lines with a thickness of 60 µm are fabricated (Figure 2b). The transmission coefficient (represented by S_21_) and loss of the lines are shown in Figure 2c,d, respectively. The loss of both the ETC and copper transmission lines increases with the frequency because of the skin effect. Besides, the loss of the printed ETC transmission line at the same frequency is comparable to that of the copper line, suggesting that printed ETC could serve as a viable alternative to copper in typical RF systems for achieving low‐loss devices.

To accurately characterize the mechanical characteristics of the ETC, a dog bone sample is printed using the ETC ink (Figure 2e). Figure 2f represents the stress–strain curves of the printed samples before and after sonication. After sonication, the EGaIn microparticles within the ETC are more uniformly dispersed, leading to a more homogeneous stress distribution and an improved fracture strain (ε_fracture_) from 316% to 460%. To measure the resistance of the ETC upon strain, a resistor sample under test is printed on a TPU substrate. Noteworthy, the resistance of the ETC is strain‐insensitive, which remains constant upon 300% strain, and slightly increases by 50% when stretched to 460% strain, as shown in Figure 2g. This behavior is different from conventional conductive composites, whose resistance changes with strain according to Pouillet's law—the ratio of the strained resistance R_ε_ to the original resistance R_0_ varies with strain ε as follows:

(1) $$\begin{array}{c} \;\frac{R_{\varepsilon }}{R_{0}}={\left(1+\varepsilon \right)}^{2} \end{array}$$

In contrast, the resistance of ETC is predominantly determined by the nanoscale EGaIn particles in the conductive network. As shown in Figure 2h, distinguished from EGaIn microparticles, these EGaIn nanoparticles well maintain their shapes upon strain, ensuring the strain‐invariant low resistance of the ETC. This limited deformation of nanoparticles is attributed to the high surface tension of EGaIn, which effectively maintains the particles' spherical shape upon strain. The surface tension becomes more significant when the partial size reduces to nanoscale due to the higher specific surface area.

### 2.3 The Design and Characterization of Stretchable Antenna

**Figure 3:**
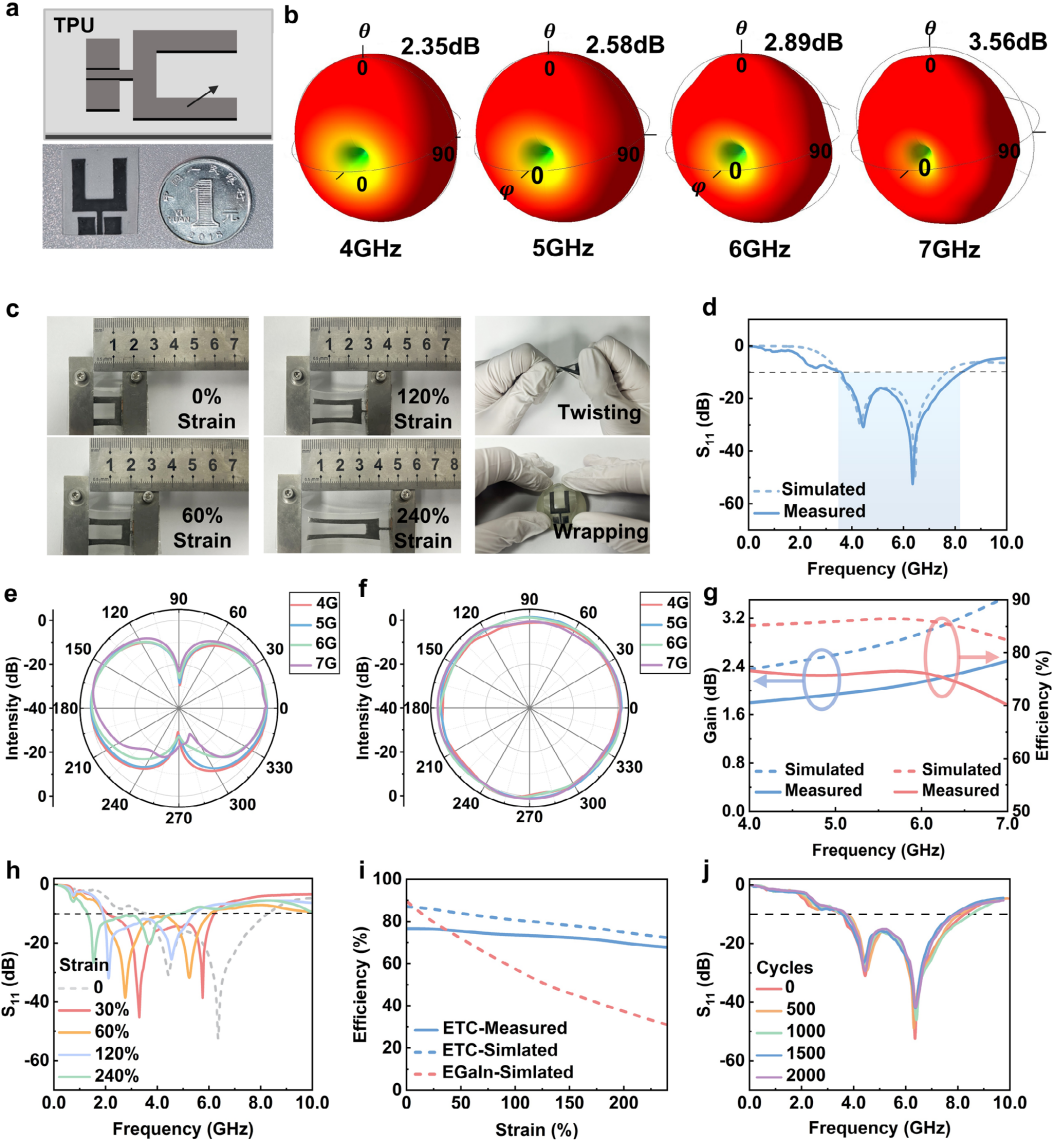
The characterization of the stretchable wideband ETC antenna. a) The schematic picture and the photograph of the antenna. b) The simulated 3‐D radiation pattern of the proposed antenna at 4, 5, 6, 7 GHz, respectively. c)The mechanical compliance of the printed antenna under twisting, wrapping, and different strains. d) The measured and simulated S_11_ of the antenna. e) The E‐plane radiation pattern of the antenna. f) The H‐plane radiation pattern of the antenna. g) The peak gain and radiation efficiency of the printed antennas. h) The measured S_11_ of the antenna upon different strains. i) The radiation efficiency of the ETC antenna and the EGaIn antenna upon different strains. j) The measured S_11_ of the antenna after different stretching cycles upon 60% strain.

Typically, planar monopole antennas are ideal for printed antenna applications due to their low profile, wide operating bandwidth, and nearly omnidirectional radiation pattern. Here, we designed an improved U‐shaped antenna by removing the middle portion of the rectangular monopole antenna (**Figure** **3**a). This design has a compact structure and reduces the material consumption of printing without distorting radiation characteristics (Figure S5, Supporting Information). Furthermore, the 3D direction patterns of the antenna remain stable and free of distortion as the frequency or strain varies, which is due to the symmetry structure that could effectively restrain the generation of the high‐order radiation modes.^[^ ^41^ ^]^ The radiation patterns of the proposed antenna maintain a donut shape from 4 to 7 GHz (Figure 3b), exhibiting omnidirectional broadside radiation traits. As shown in Figure 3a, the designed U‐shaped antenna is directly printed on a TPU dielectric substrate and fed through a CPW structure. The printed antenna could be easily stretched, twisted, and wrapped around other objects without any physical fractures (Figure 3c).

The operating bandwidth refers to the frequency band where the antenna's reflection coefficient (represented by S_11_) is lower than −10 dB. As illustrated in Figure 3d, the measured S_11_ is lower than −10 dB from 3.75 to 8.21 GHz (a fractional bandwidth of 75%), indicating the antennas could efficiently radiate or receive signals. The radiation pattern of the printed antenna can be described by its E‐plane and H‐plane patterns. The E‐plane is the plane containing the electric‐field vector and the direction of maximum radiation while the H‐plane is the plane containing the magnetic‐field vector and the direction of maximum radiation. The measured radiation pattern of the antenna demonstrates the expected broadside radiation. Figure 3e,f represents that the directional pattern on the E‐plane appears a figure‐of‐eight shape, while it appears a circular pattern on the H‐plane, meeting the requirements of prevalent wireless communication antennas.^[^ ^42^ ^]^ The peak gain and radiation efficiency of the antenna are shown as the blue lines and orange lines in Figure 3g, respectively. On the one hand, the measured peak gain increases with the frequency, consistent with the simulation results. This increase is due to the stronger directivity (higher peak gain in a specific direction) of the antenna at a higher frequency. Besides, the peak gain of the antenna is higher than 1.8 dB, comparable to a typical omnidirectional antenna.^[^ ^43^ ^]^ The deviation between the simulation and measured result (≈0.5 dB) is primarily attributed to extra loss introduced by packaging and coaxial cable. On the other hand, radiation efficiency is defined as the ratio of radiated power to input power, which is determined by ohmic loss and substrate loss, and can be calculated by the following equation:

(2) $$\begin{array}{c} \eta =\frac{P_{input}-P_{ohm}-P_{sub}}{P_{input}} \end{array}$$

where *P_input_*,*P_ohm_*,*P_sub_* are the power input into the antenna, ohmic loss power, and substrate loss power, respectively. Thanks to the high conductivity of the ETC, the antenna achieves a radiation efficiency exceeding 70% across its entire operating bandwidth, which is higher than that of most previous strategies such as graphene (64.9%), Ag Nanowire (AgNW) (30.0%), and 3D‐assembly‐copper‐foil (60.1%).^[^ ^10^, ^44^, ^45^ ^]^

As a stretchable antenna, it is important to assess the effect of mechanical deformations on the RF performance. The S_11_ parameter is measured as the strain varies from 0% to 240%. It can be seen from Figure 3h that the operating frequency shifts toward lower frequencies as the antenna is stretched, aligning with theoretical predictions. Notably, thanks to the wide‐band characteristics of the designed antenna, the operating frequency still covers from 2.4 to 5.1 GHz (covering N77, N78, and N79 bands in IEEE 802.11a protocol) upon 240% strain. This result implies that the printed antenna could transmit and receive signals in a specific communication band even upon large strain, which is supported in later demonstration. Moreover, because of the strain‐invariant resistance of the printed ETC antenna, the ohmic loss of the antenna remains low upon strain. As a result, the antenna maintains high radiation efficiency upon strain, consistent with the simulation results (Figure 3i). By contrast, the simulated result shows that the same‐size antenna fabricated by pristine EGaIn exhibits a radiation efficiency of only 31% upon 240% strain, representing a 56% decrease from its peak efficiency of 87%.

For a robust stretchable communication device, it's essential to survive upon strain cyclability tests. Therefore, the performance of the printed antenna is measured as a function of the number of stretching cycles. The antenna is subjected to 500, 1000, 1500, and 2000 times upon 60% strain, and the results are shown in Figure 3j. Owing to the self‐healing characteristic of the ETC (Figure S6, Supporting Information), the measured S_11_ shows negligible changes after cyclic strain, validating the robustness of the printed antenna. Moreover, the antenna also shows excellent robustness under high temperature and water bath states (Figure S7, Supporting Information).

**Figure 4:**
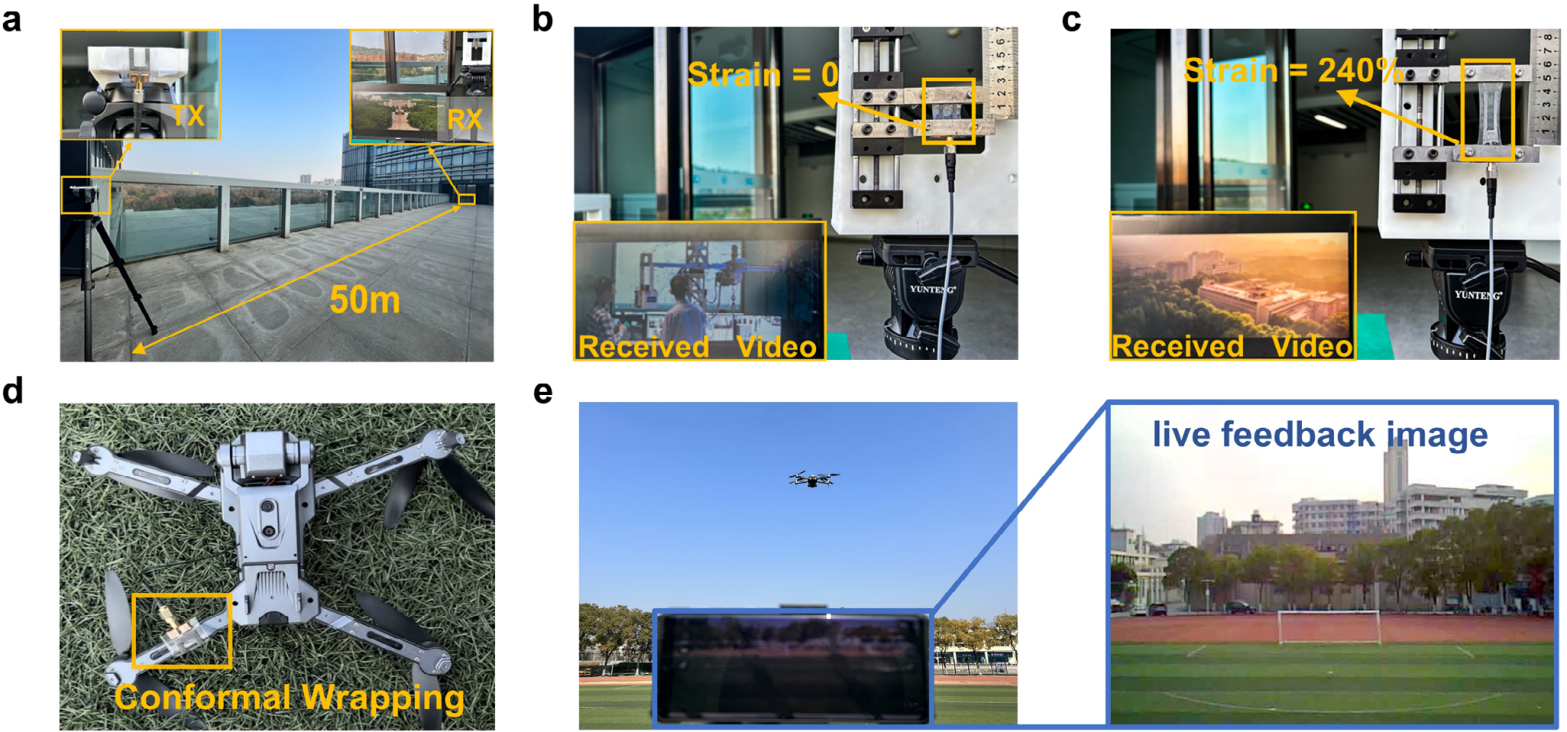
The communication demonstration of the wideband stretchable ETC antenna in a terrestrial system and an airborne system. a) The demonstration set‐up of wireless video transmission experiment. b) The photograph of the antenna transmitting video before strain c) The photograph of the antenna transmitting video at 240% strain. d) The photograph of the antenna conformally wrapped around the drone for real‐time wireless image transmission. e) The real‐time wireless image transmission experiment using the printed antenna under bent state.

Stretchable antennas have wide application scenarios, particularly in real‐time wireless communication. As a proof‐of‐concept demonstration, the printed stretchable antenna is tested for video‐signal transmission in an open field. As depicted in **Figure** **4**a, two printed antennas are mounted on brackets 50 meters apart, with one antenna transmitting video signals at an input power of ‐5dBm and the other receiving them. Both antennas operate at a communication frequency of 4.9 GHz, compliant with the 5G communication protocol. As shown in Figure 4b and Video S2 (Supporting Information), the video is successfully received. Then the receiving antenna is stretched from 0% to 240% strain and the video remains continuous (Figure 4c; Video S3, Supporting Information), showing robust communication quality upon strain. Besides static data communication, an airborne mobile communication experiment is conducted. The antenna is bent and conformally wrapped around the drone (Figure 4d), transmitting real‐time images to the operator at a frequency of 5.8 GHz. The image transmission remains smooth during the flight that reaches a height of 15 meters and a maximum vertical distance of ≈50 meters (Figure 4e; Video S4, Supporting Information). These results further confirm the potential of printed stretchable antennas in diverse robust wireless communication applications.

## 3 Discussion

In summary, we present a universal EGaIn‐based DIW printing strategy for fabricating wideband stretchable antennas. The printed antenna achieves an operating bandwidth of 3.75–8.21 GHz (fractional bandwidth: 75%), a peak gain of 1.8 dB, and a radiation efficiency of 76.6% surpassing state‐of‐the‐art stretchable antennas listed in Table S1 (Supporting Information). More importantly, the antenna maintains stable wireless communication even under 240% strain and conformal wrapping.

This progress results from the synergistic optimization of materials and printing processes. The TPU‐modified ink exhibits shear‐thinning behavior (viscosity drop from 153 to 5 Pa s at shear rates > 100 s⁻¹), ensuring clog‐free extrusion through 10 µm nozzles and maintaining high print fidelity. Unlike conventional EGaIn inks that depend on microfluidic confinement, our ink composite enables self‐supported patterning via controlled surface energy (contact angle: 86.9° vs 141.3° for pristine EGaIn), ensuring stable stacking during DIW printing. These rheological and surface modifications are crucial for achieving both high‐resolution and large‐area conductive patterns.

Different from other LM composites that require mechanical activation (e.g., stretching or sintering) to break the oxide shell and establish conductive pathways, our TPU‐modified ink achieves conductivity via ultrasonic activation (Table S1, Supporting Information). This process restructures EGaIn microparticles into smaller nanoparticles (∼400 nm) without rupturing the oxide layer, ensuring both mechanical robustness and high conductivity (1.6 × 10⁶ S m^−1^). The increased surface area of rearranged EGaIn particles significantly reduces the risk of leakage (Figure S8, Supporting Information). Furthermore, the smaller EGaIn particles form a more uniform and densely packed conductive network, improving stress distribution and preventing localized stress concentrations. This structural enhancement is further corroborated by tensile testing, where the ultrasonically treated samples exhibit a higher elastic modulus and fracture strain, indicating improved mechanical robustness and reduced leakage potential.

We also compared the printed ETC antenna with the state‐of‐the‐art schemes of stretchable far‐field antennas (Table S2, Supporting Information). The printed ETC antenna exhibits superior RF and mechanical performance owing to the intrinsic properties of the ETC. For instance, it shows higher radiation efficiency than other antennas based on AgNW and Graphene due to higher conductivity. Besides, the ETC composite showed remarkable resistance stability under the cyclic strain tests, with less than 50% resistance increase at 460% strain, significantly outperforming AgNW composites (600% increase at 40% strain). The TPU matrix in our composite prevents structural fractures, while the ETC composite demonstrates exceptionally low strain‐induced efficiency loss (SEL: 0.0375%) and ensures excellent RF performance even under high strain, outperforming both graphene (which suffers from bond fracture issues) and AgNW (SEL: 0.667%). This combination of low SEL and high fracture strain gives the ETC antenna outstanding stretchability without sacrificing RF performance, far exceeding that of Ag ink, copper, and pristine EGaIn‐based antennas.

Besides the demonstrated applications, the EGaIn‐based DIW printing strategy could also benefit many other applications. For example, the proposed strategy could be used to realize reconfigurable intelligent surfaces due to the high conductivity and flexibility of the ETC, improving its complex and costly deployment. In addition, the modulation of electromagnetic parameters through stretching offers a novel type of reconfigurable RF devices, such as filters, RF chokes, and antennas. In a word, the proposed scheme paves the way for further active applications of stretchable electronics.

## 4 Experimental Section

### Materials

Gallium and indium were purchased from Hebei Jiuyuexin Materials Technology. TPU (SP‐80A) was received from Lubrizol LifeSciences Tecophilic. The polar groups of TPU interact with the surface oxide layer of EGaIn, which helps to improve the uniform distribution of EGaIn in the matrix. The SP series TPUs were designed for solution processing, offering excellent solubility in organic solvents. Among the available SP grades (SP‐80A, SP‐93A, SP‐60D), SP‐80A was chosen due to its Shore hardness of 70A (softer than SP‐93A and SP‐60D) and its superior elongation at break (1000%), making it well‐suited for flexible and stretchable applications. DMAC was purchased from Energy Chemical, which was chosen for its high dissolving capacity with TPU, low evaporation rate under ambient conditions, and low toxicity. These properties make DMAC the preferred solvent over alternatives such as Dimethylformamide (DMF), N‐Methyl‐2‐Pyrrolidone (NMP), and Tetrahydrofuran (THF). Acetone was used as a dispersant and purchased from Sinopharm Chemical Reagent.

### Experimental Details of the DIW System

The DIW system was composed of a nozzle (Dongguan Yidizhan, YDZ‐LK.34G‐6MM‐100) connected to an ink reservoir, a pneumatic liquid dispenser (Nordson, Ultimus V), a motion controller (Leadshine, SMC606), and a three‐axis mobile platform with automatic movements in the x, y, or z axis (Figure S9, Supporting Information). The nozzle was composed of a stainless‐steel needle tip (inner diameter: 60 µm; outer diameter: 160 µm; length: 6 mm) for smooth ink flow and a plastic hub for secure mounting and reduced weight. The nozzle underwent a two‐step cleaning process before printing, including an ultrasonic alcohol bath to remove residual particles and a plasma treatment to minimize ink adhesion on the inner walls, ensuring reliable extrusion.

The nozzle with an ink reservoir was mounted to the z‐axis positioning arm and a TPU substrate was placed on the horizontal platform. The distance between the nozzle tip and the substrate was controlled to be 50–100 µm, and the dispenser applied pneumatic pressure (≈600 kPa) to extrude the ink onto the substrate through the nozzle. The nozzle moved along a rasterized toolpath with a velocity of 0.8 mm s^−1^ and extruded a series of parallel ink lines to print the predetermined composite pattern. The gap between two adjacent lines was set to 50 µm.

### Preparation of Ink and Conductive Composite

Gallium was alloyed with indium to form EGaIn (eutectic gallium indium, 75% Ga, 25% In, by weight) resulting in a melting point of ≈15.5 ^○^C. EGaIn was then emulsified by an ultrasonic cell disrupter (Ningbo Keshen, KS‐500F). A centrifuge tube containing 30 ml of acetone was used as a dispersant. Then 2 g of EGaIn was added to the tube and emulsified into microparticles by the sonication probe. The sonication lasted 30 min at a power of 270 W (54%). The emulsion was unstable after sonication and the high density of EGaIn makes it settle after 2 h even microparticles. The sediment of microparticles was then mixed with the TPU dissolved in a DMAC solution (400 mg mL^−1^) using a planetary mixer (THINKY, ARM‐310) set to 2000 rpm for 10 min, forming the ETC ink. The ETC ink was then used in the DIW system and printed into a customized pattern in its liquid state. Subsequently, the printed pattern was heated in a drying oven at 60 °C for 4 h to ensure complete evaporation of the DMAC solvent. After heating, DMAC was fully removed, and the printed pattern was completely dried (Figure S10, Supporting Information) and solidified. The solidified printed pattern was then immersed in water at a depth of 10 cm, and an ultrasonic probe (power: 325 W) was positioned 3–5 mm above the sample to activate the printed pattern through a 30 s sonication process.

### The Characterization of the ETC Ink

Contact angles of EGaIn ink and the ETC ink were measured using the sessile drop method. The SEM image was characterized by scanning electron microscopy (Nova NanoSEM 450 FP2053/45). The trace width was measured using a metallurgical microscope (Sunny Optical Technology, RX50M) with a 50X microscope objective, and thickness was measured by a stylus profilometer (Bruker, DektakXT). The conductivity was measured by the Four‐Probe Method. The samples were printed as square films with dimensions of 20 mm × 20 mm × 20 µm (length × width × height) for conductivity measurements. The four‐probe testing method necessitates a rigid substrate to avoid measurement errors caused by mechanical deformation during testing. Therefore, samples for electrical conductivity measurements were directly printed on glass substrates to minimize measurement errors. The sample was then placed on the Four‐Probe test bench (Guangzhou 4Probes Tech, S‐2A). A source meter (Keithley, 2400 SourceMeter) output voltage and detected current through a Four‐Probe (Guangzhou 4Probes Tech, FT‐203).

The sheet resistance *R_s_* calculated by the four‐point probe method is given by the following equation:

(3) $$\begin{array}{c} \;R_{s}=\frac{V}{I}\;\times \frac{\pi }{\ln2} \end{array}$$

where *V* is the measured voltage (in V) and *I* is the applied current (in A).

Then, the conductivity σ is calculated from the sheet resistance *R_s_*:

(4) $$\begin{array}{c} \sigma =\frac{1}{Rs\times t} \end{array}$$

where *t* is the thickness of the film, which is further confirmed by cross‐sectional microscopy images of the printed pattern (Figure S11, Supporting Information).

All mechanical stress–strain tests were measured by a Materials Testing Machine (Shenzhen Suns, UTM2203). The resistances under different strains were recorded by a multimeter (Victor, VC890C). The tensile fracture experiment sample was printed onto a glass substrate to form dog‐bone‐shaped patterns (central length: 12 mm; width: 3 mm).

### The Measurement of the Attenuation Constant

For the electromagnetic test, the sample was printed as a coplanar waveguide (Figure S12, Supporting Information). The reflection coefficient (S_11_) and the transmission coefficient (S_21_) of the copper line and ETC line were measured by a vector network analyzer (VNA, Keysight N5227B), and their attenuation constants α were calculated by the following equation:

(5) $$\begin{array}{c} \alpha =\frac{1}{l}\;10\mathrm{lg}\left(\frac{1-{\left|S_{11}\right|}^{2}}{{\left|S_{21}\right|}^{2}}\right) \end{array}$$

where *l* is the length of the line.

### The Measurement of the Stretchable Antenna

To minimize the electromagnetic wave scattering from the test environment, the RF performances of the printed antenna including operating bandwidth, radiation pattern, peak gain, and radiation efficiency were all measured in the microwave anechoic chamber at Huazhong University of Science and Technology (Figure S13, Supporting Information).

The S_11_ of the AUT was measured using VNA (Keysight N5227B). The VNA was calibrated first, and then the AUT was connected to it through a feed connector. The scanning range of the VNA was set from 10 KHz to 10 GHz. Two frequency points *f_h_* and *f_l_* with S_11_ of −10 dB were recorded, respectively, and the fractional bandwidth *B_f_* of the antenna was calculated by Equation S4 (Equation S4, Supporting Information).

The far‐field radiation pattern was measured by an antenna measurement system. The AUT was fixed at the rotational table and separated the standard gain horn antennas by a certain distance. The scanning angle of the system was set at a 2° interval in the scanning range −180°–180° and the scanning was set for scanning in only principal planes (E and H). The ESA (JiujinTech PSA5000A) was used to record the measured intensity of the electric field at each angle.

The gain of the printed antenna was measured by the gain‐comparison method. Two standard gain horn antennas (HD‐48/58SGAH15) were connected to the microwave source (Agilent E8247C) and the ESA, respectively. The gains of the standard gain horn antennas were known from the antenna manual provided by antenna manufacturers. The output power of the ESA was set at 10dBm. The received power of the standard gain horn antenna and the printed antenna were recorded, respectively. The gain of the printed antenna was calculated by Equation S5 (Equation S5, Supporting Information).

## Conflict of Interest

The authors declare no conflict of interest.

## Author Contributions

X.G. and Y.L. contributed equally to this work. Y.Y., M.S. conceived the idea and supervised the project. X.G. designed the project. Y.L. carried out the device fabrication. X.G. and Y.L. wrote the manuscript. X.G., Y.L., H.P., and Z.Z. performed the device measurement. Y.Y., M.S., and Y.G. helped in revising the manuscript. All authors contributed to analyzing the data.

## Supporting information



Supporting Information

Supplemental Video 1

Supplemental Video 2

Supplemental Video 3

Supplemental Video 4

## Data Availability

The data that support the findings of this study are available from the corresponding author upon reasonable request.
