# Supplementary material for: Direct‐Ink‐Writing Printed Stretchable Eutectic Gallium–Indium Antenna for Robust Wireless Communication
Source: Adv Sci (Weinh). 2025 Apr 2;12(21):2414285. doi: 10.1002/advs.202414285 (PMC12140361; doi:10.1002/advs.202414285)
Supplement: Supplementary file 1 — Supporting Information [file ADVS-12-2414285-s003.docx]

**Supporting Information**

**Direct-Ink-Writing Printed Stretchable Eutectic Gallium-Indium** **Antenna for Robust Wireless Communication**

**Table of contents**

**Supplementary Table**

**Table S1** Comparison of the EGaIn patterning strategy for conductive inks.

**Table S2** Comparison of the state-of-the-art stretchable far-field antennas.

**Supplementary Figures**

**Figure S1** Raster printing of the formulated ink.

**Figure S2** Raster printing of the formulated ink.

**Figure S3** Photographs of ETC lines printed with a 10 µm inner-diameter nozzle.

**Figure S4** Scanning electron microscopy (SEM) image of the ETC.

**Figure S5** Configuration and simulation of antenna.

**Figure S6** Self-recovery of ETC line.

**Figure S7** Robustness of stretchable antennas in challenging environments.

**Figure S8** Comparison of samples’ resistance before and after ultrasound.

**Figure S9** DIW system composed by a three-axis motion platform.

**Figure S10** The thermogravimetric analysis of ETC

**Figure S11** The cross-section microscopy image of the ETC line.

**Figure S12** The schematic diagram of the coplanar waveguide structure.

**Figure S13** The photograph of the experimental set-up in an anechoic chamber.

**Figure S14** Photograph of ETC’s resistance measurements using a multimeter.

**Figure S15** Tensile test of the ETC.

**Table S1: Comparison of the EGaIn patterning strategy for conductive inks**

| Strategy | | Activation | Conductivity (S/m) | Average Particle  Size | Leakage  Issue |
| --- | --- | --- | --- | --- | --- |
| Pristine EGaIn | microfluidics^[1]^ | \ | 3.4*10^6^ | \ | Y |
| EGaIn Composite | EGaIn-Cu Particle ^[2]^ | \ | 6 × 10^6^ | \ | Y |
|  | EGaIn-Fe Particle ^[3]^ | \ | 1.53 × 10^6^ | \ | Y |
|  | EGaIn-Polymer | mechanical sintering^[4]^ | \ | 1μm | Y |
|  |  | strain^[5]^ | 2.5×10^5^ | 3 μm | Y |
|  |  | sonication  **(This work)** | 1.6*10^6^ | 400 nm | N |

**Table S2:** **Comparison of the state-of-the-art** **stretchable far-field antennas**

| Number | Material | Fabrication | Conductivity  (S/m) | Ultimate strain | Bandwidth | Radiation efficiency | SEL* | | Communication demonstration |
| --- | --- | --- | --- | --- | --- | --- | --- | --- | --- |
| 1^[6]^ | Ag flakes ink | Mask printing | \ | 30% | 2.1-2.7  GHz | \ | | \ | Y |
| 2^[7]^ | Ag flakes ink | Mask printing | 1*10^6^ | 15% | 800-980 MHz | 80% | | 1.13% | Y |
| 3^[8]^ | Copper-Serpentine | Laser patterning | 5*10^7^ | 25% | 2.40–2.48 GHz | \ | | \ | Y |
| 4^[9]^ | Copper-Serpentine | Circuit etching | \ | 30% | 2.2-3.4  GHz | \ | | \ | Y |
| 5^[10]^ | Copper-3D assembly | Circuit etching | \ | 30% | 1.00-1.15 GHz | 62% | | 0.900% | N |
| 6^[11]^ | AgNW | Spin  coating | 7*10^5^ | 40% | 2.25-2.45 GHz | 30% | | 0.667% | N |
| 7^[12]^ | AgNW | Mask printing | 8.1*10^5^ | 15% | 2.8-3  GHz | 40% | | \ | N |
| 8^[13]^ | Graphene | Screen Print | 7*10^3^ | \ | 1.7-5  GHz | 60% | | \ | N |
| 9^[14]^ | EGaIn | Microchannel | 3.4*10^6^ | 40% | 3-11  GHz | 90% | | 0.375% | N |
| 10^[15]^ | EGaIn | Microchannel | 3.4*10^6^ | \ | 3.4-3.5  GHz | 40% | | \ | N |
| 11^[16]^ | Ag  composite | Mask printing | 8*10^4^ | 20% | 2.34-2.62  GHz | \ | | \ | N |
| 12 (this work) | ETC | DIW | 1.6*10^6^ | >240% | 3.75-8.21 GHz | 76.6% | | 0.0375% | Y |

* The Strain-Efficiency Loss (SEL) quantifies the average decrease in antenna radiation efficiency per 1% of strain at the maximum tested deformation.


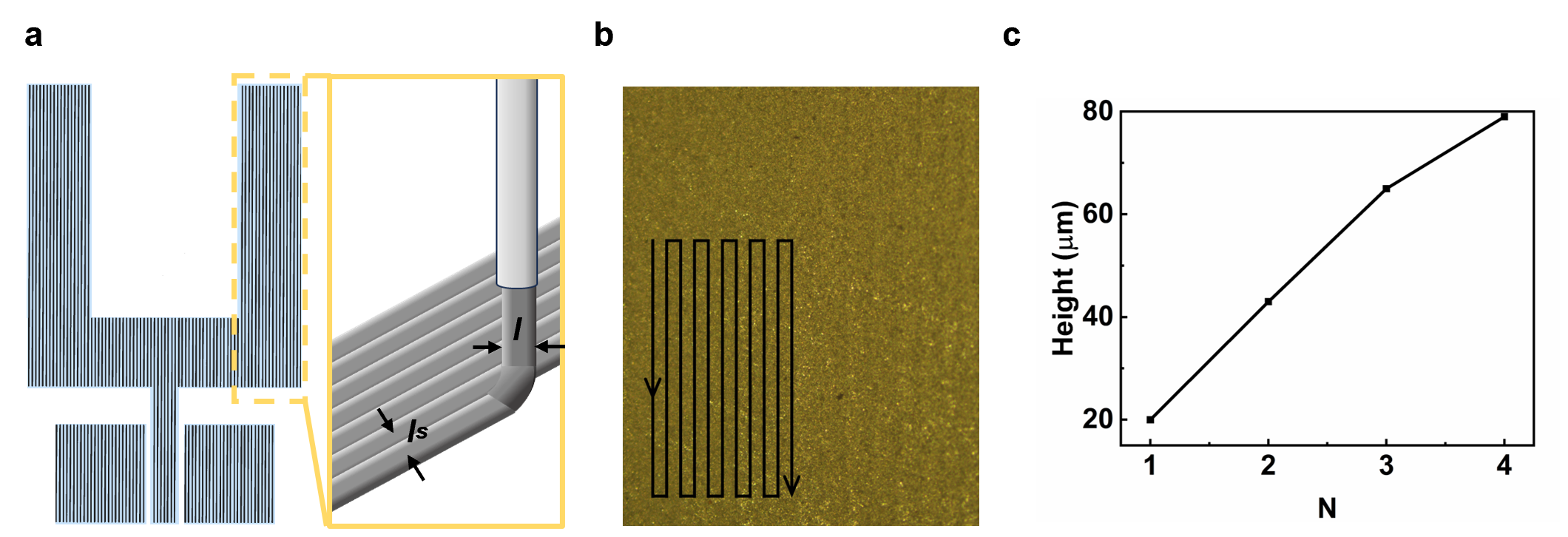


**Figure S1** Raster printing of the formulated ink. (a) The schematic diagram of stacking lines to a layer. (b) The optical microscopy images of printed smooth layer traces after stacking. (c) Thickness plotted as a function of layer number.

Extrusion and stacking are the key steps in the DIW printing process. The extrusion utilizes shear-thinning properties to achieve smooth ink extrusion of ink through the nozzle. The stacking process relies critically on the ink’s rapid recovery to a high viscosity after extrusion to prevent deformation.

“Stacking lines to a layer” means precisely aligning and partially overlapping successive lines to construct highly customized two-dimensional conductive patterns. During this process, ink is extruded through a nozzle under controlled air pressure, forming lines of width l on the substrate. To ensure 2D conductive patterns, adjacent lines are printed with a spacing *ls* smaller than the line width *l* (*ls* < *l*) ^[17]^(Figure S1a). This overlap compensates for potential gaps caused by ink shrinkage during heating. The optical microscopy images of printed smooth layer traces after stacking are illustrated in Figure S1b, and the thicknesses of the printed traces are plotted as a function of layer number (Figure S1c).


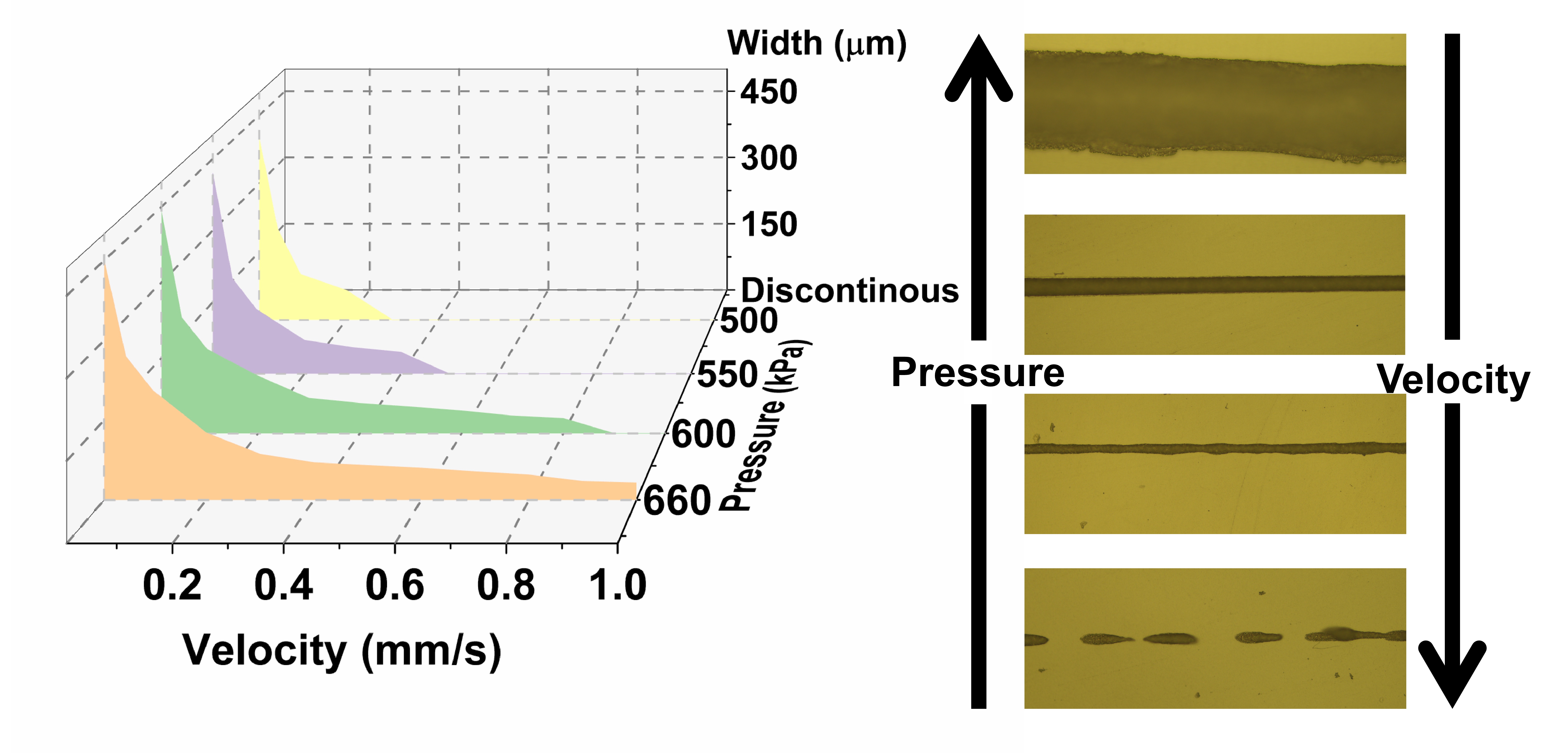


**Figure S2** Printing parameter testing for formulated EGaIn-TPU ink.

The mono-fiber printing experiment is used to verify how to control the basic printing parameters to ensure the stability and continuity of the printed EGaIn-TPU composite (ETC) fiber. In this paper, a needle with *D*=0.06 mm and a print height of *H*=100 um was used as the basis for printing experiments. Multiple combinations of air pressure *P* and print speed *V* can usually be found for a selected needle diameter. Considering the printing accuracy and printing efficiency, this paper sought the best printing speed V under several cases of air pressure. As the printing speed increased and the input air pressure decreased, the single fiber formed several different shapes: coarse fibers with rough edges deviating from printing accuracy, straight fibers with clear edges, and discontinuous fibers. Through multiple printing trials, we found that lines with a width close to the needle's inner diameter (60 µm) had the clearest edges. At an air pressure of 660 kPa, the clear-edged lines with a width of approximately 60 µm are printed at a wide range of speed.


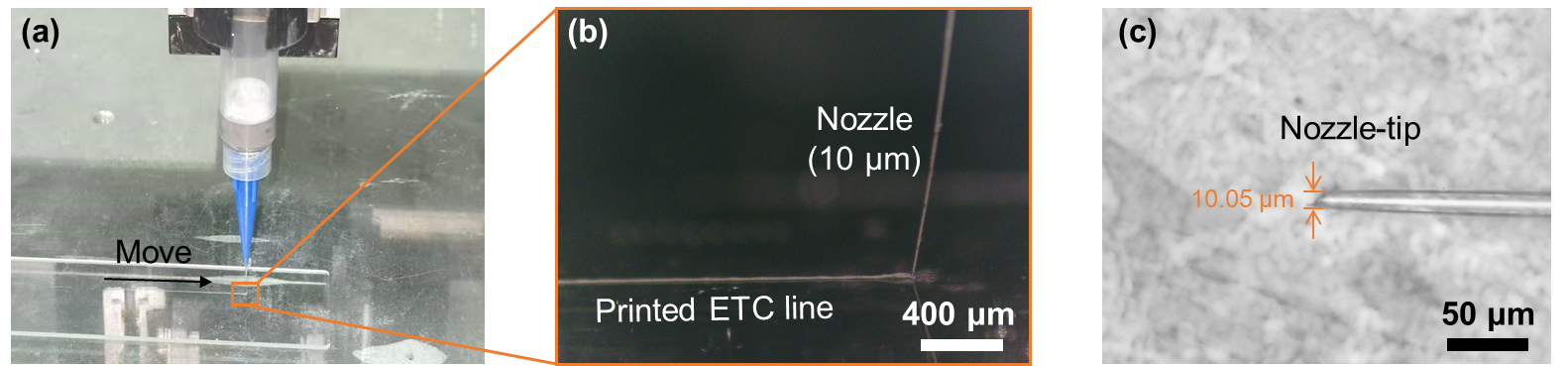


**Figure S3** (a) Photograph of ETC lines printed with a 10 µm inner-diameter nozzle. The black arrow indicates the nozzle movement direction, demonstrating continuous printing over a large area. (b) Magnified CCD image near the nozzle tip, showing highly uniform lines with no interruptions or clogging during extrusion. (c) The microscopy image of the nozzle.


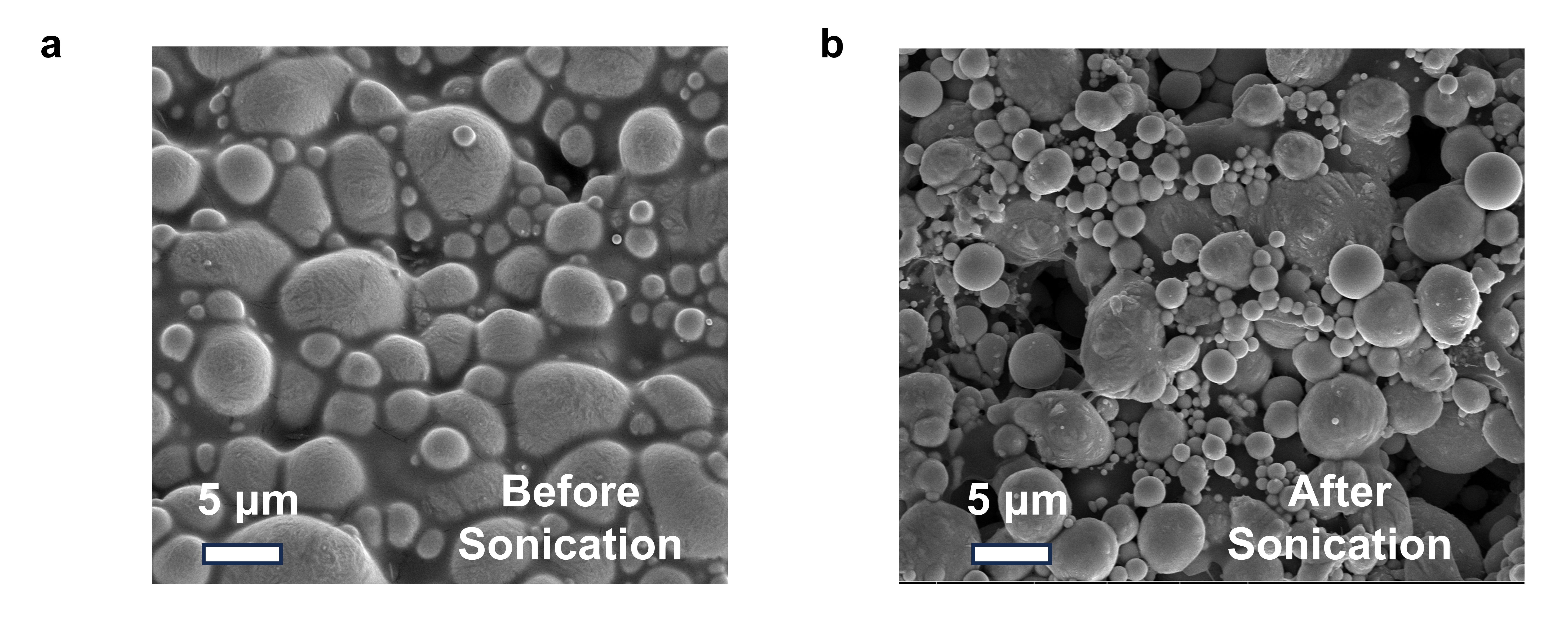


**Figure S4** Scanning electron microscopy (SEM) image of the ETC before (a) and after (b) sonication

The enhancement in conductivity of secondary sonication is attributed to the formation of extra-conductive pathways. ETC samples (the content of EGaIn is 75 vol%) are printed and observed through SEM images, Figure S4a shows that the EGaIn microparticles before sonication show an average size of ~2 μm, and they are isolated from each other. After sonication, many EGaIn microparticles fracture into smaller nanoparticles with an average size of 400 nm, as illustrated in Figure S4b. These smaller nanoparticles are dispersed between the isolated large EGaIn microparticles and form conductive pathways.


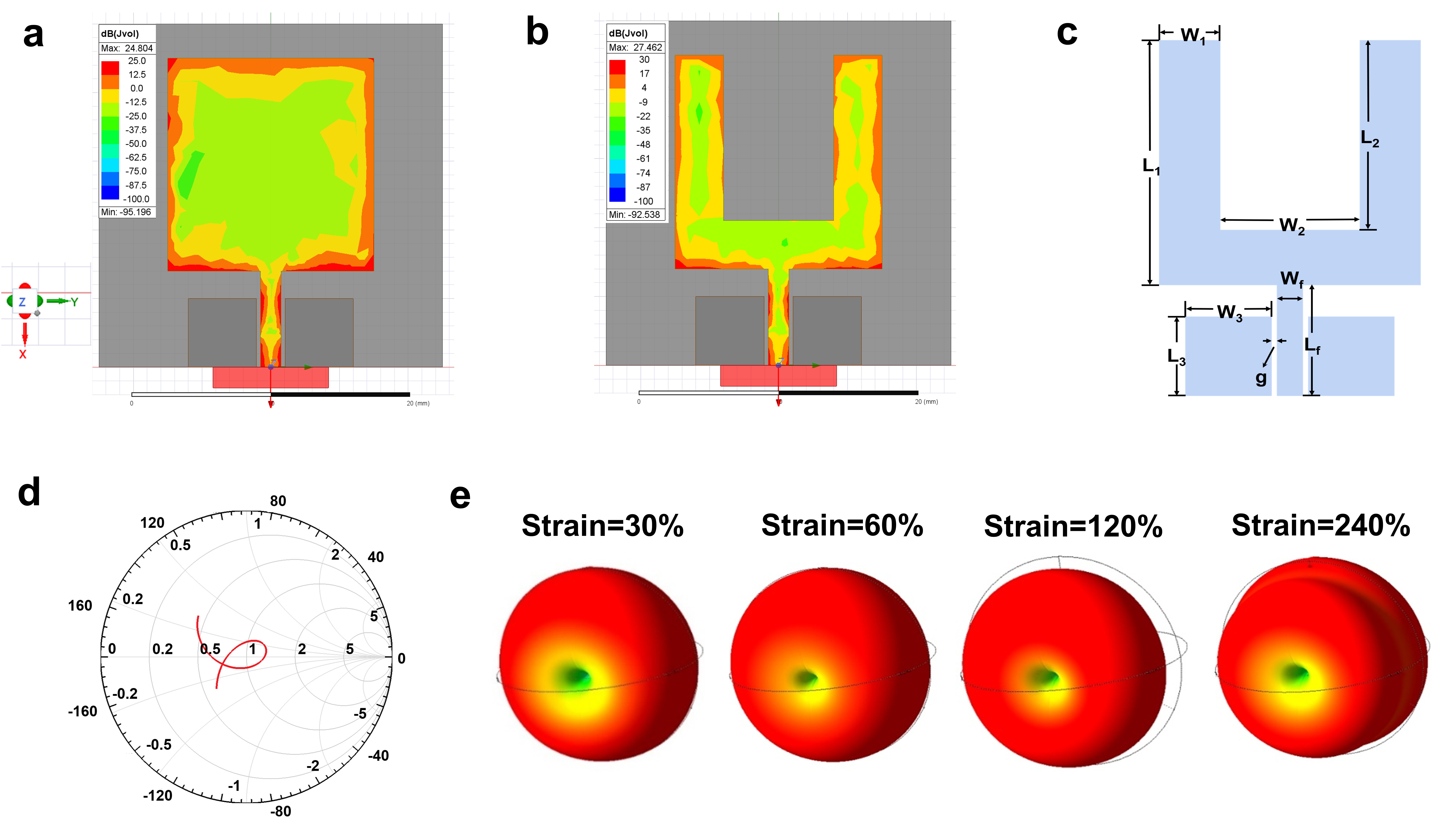


**Figure S5** The design of the proposed antenna. (a) Current density distribution of rectangular monopole antenna. (b) Current density distribution of the modified monopole antenna. (c) The proposed antenna configuration. (d) Simulated Smith chart from 3 GHz to 8 GHz. (e) The simulated radiation patterns of the antenna under strain.

Typically, planar monopole antennas are ideal for printed antenna applications due to their low profile, wide operating bandwidth and nearly omnidirectional radiation pattern. However, the traditional monopole antenna (such as rectangular patch antennas) has a large footprint and makes the printing material-consuming. To address this, we have modified the design of the monopole antenna. Considering that the electric current primarily flows along the outer edges of monopole antennas, removing the central section has a minor effect on the radiation performance (Figure S5a and Figure S5b). Thus, the planar monopole antenna is modified by removing its central portion in our work, resulting in a simpler structure resembling a "U-shape antenna" (Figure S5c). Since the radiation frequency is determined by the total length of the antenna, the U-shaped structure—with two arms effectively extending the length, can achieve the desired frequency while maintaining a more compact footprint.^[18]^

The specially designed U-shape structure of the proposed antenna makes the reactance insensitive to the frequency and thus has a wide operating bandwidth theoretically. The simulated Smith chart (Figure S5d) shows a great impedance matching between 3 GHz to 8 GHz. The curve near the center of the chart indicates that the resistance of the antenna is close to 50 ohms, while the reactance is about 0, implying a broad operating bandwidth.

Moreover, the 3D direction patterns of the antenna remain stable and free of distortion as the frequency or strain varies, which is due to the symmetry structure that could effectively restrain the generation of the high-order radiation modes (Figure S5e).^[19]^ The central operating frequency $f_{0}$ could be calculated by the following equations:

$$\begin{aligned} f_{o}\approx\frac{c}{\sqrt{\varepsilon_{eff}}\times L_{e}}\#\left( S1 \right) \end{aligned}$$

Where the $c$ is the velocity of light in a vacuum and the $L_{e}$ is the estimated current path of the radiating patches, i.e. along the outer edges of the designed antenna. The $\varepsilon_{eff}$ is the effective dielectric constant. The $L_{e}$ and the $\varepsilon_{eff}$ are calculated as follows:

$$\begin{aligned} L_{e}=L_{1}+\frac{W_{2}}{2}+W_{1}\#\left( S2 \right) \end{aligned}$$

$$\begin{aligned} \varepsilon_{eff}=\frac{\varepsilon_{r}+1}{2}+\frac{\varepsilon_{r}-1}{2}{(1+12\frac{h}{w})}^{-\frac{1}{2}}\#\left( S3 \right) \end{aligned}$$

Where the $\varepsilon_{r}$ is the relatively dielectric constant of the substrate material. $w$ and $h$ are the width and thickness of the substrate, respectively. $L_{1}{,W}_{1},W_{2}$ are the structural parameters of the proposed antenna and can be seen in Figure S5c.

Two frequency points $f_{h}$ and $f_{l}$ with S_11_ of -10 dB were recorded respectively, and the fractional bandwidth $B_{f}$ of the antenna was calculated as follows:

$$\begin{aligned} B_{f}=\frac{f_{h}+f_{l}}{2\times{(f}_{h}-f_{l})}\#\left( S4 \right) \end{aligned}$$

The gain of the printed antenna ($G_{AUT}$) was calculated by the following equation:

$$\begin{aligned} G_{AUT}=G_{std}+\left( E_{AUT}-E_{std} \right)\#\left( S5 \right) \end{aligned}$$

Where *E_std_* and *E_AUT_* are receive power of the standard gain horn antenna and the printed antenna, respectively. *G_std_* is the gain of the standard gain horn antennas


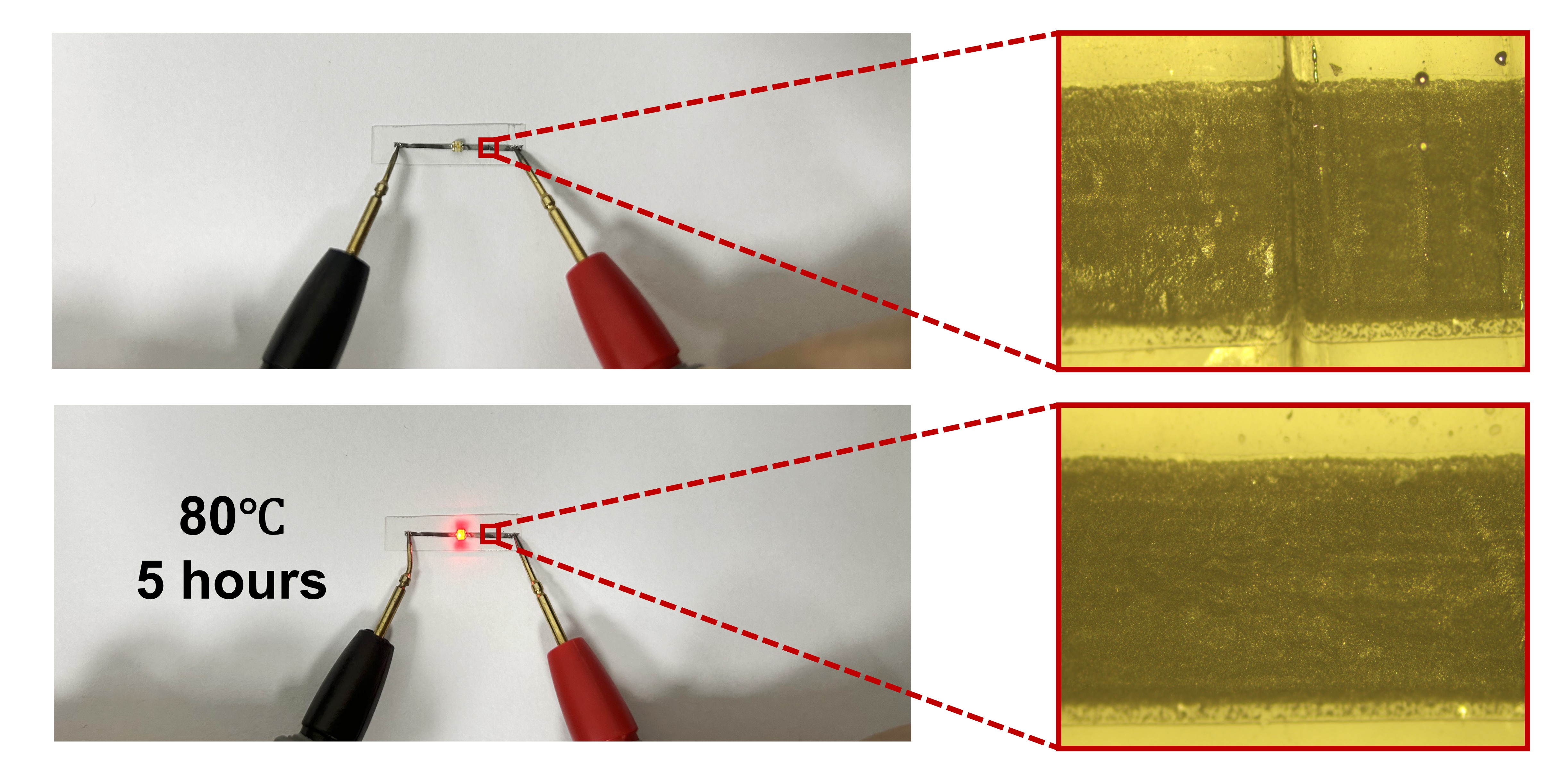


**Figure S6** Self-recovery characteristic of ETC line.

The ETC line could autonomously restore its electrical properties when damaged. The photograph (left) and optical microscopy images (right) of a damaged and self-recovered ETC line are shown in Figure S6. Heating at 80°C for 5 hours induces thermal motion and hydrogen bond reformation, leading to re-polymerization of the fractured TPU molecules and restoration of conductivity in the damaged conductor.


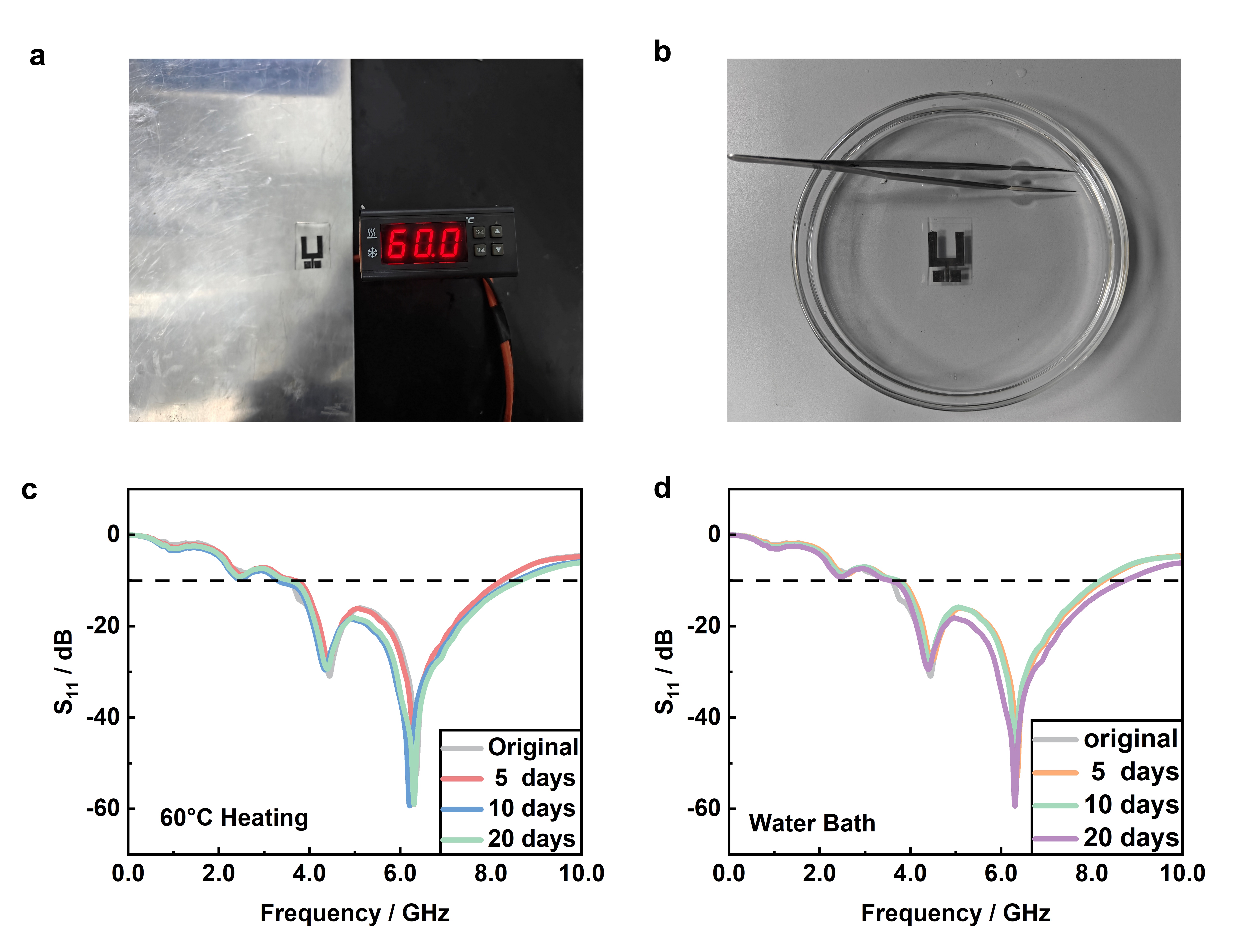


**Figure S7** Robustness of Stretchable Antennas in Challenging Environments. (a) The antennas under heating. (b) The antennas exposed in water. The S_11_ of EGaIn-PU composite antenna after different days of heating (c) and water bath (d).

To further evaluate the antenna's robustness in more challenging conditions, the radiation performances under harsh environments are characterized. The 60° heating table and a water bath are built to imitate the environments of high temperature and high humidity, respectively. Then the antennas are exposed in them for a few days, as shown in Figure S7a and Figure S7b. As depicted in Figure S7c and Figure S7c, the S_11_ merely shows slight change after 20 days’ exposure. In other words, the antenna could survive in different scenarios (such as ironing and washing) over extended periods without sacrificing its performance.


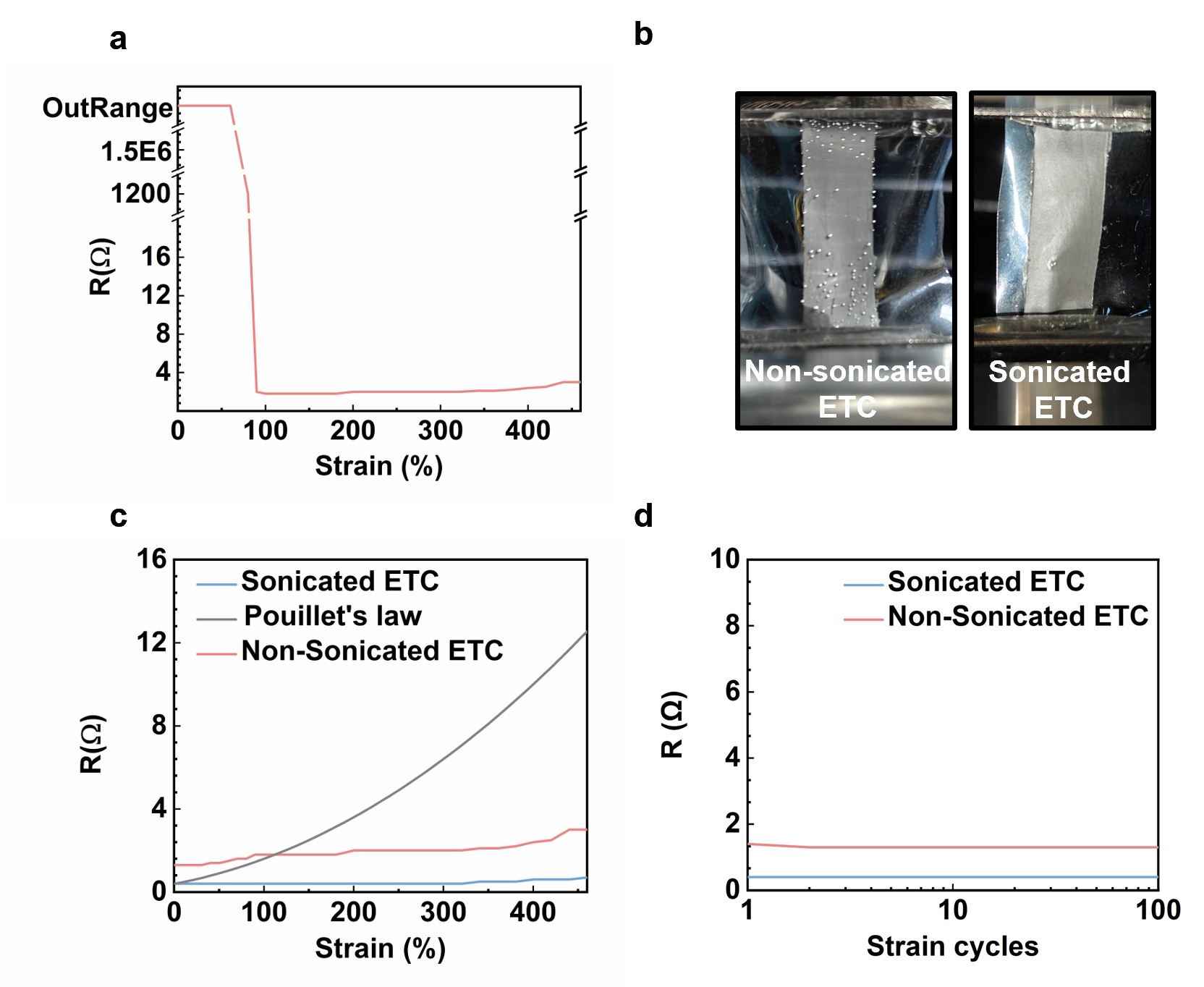


**Figure S8** (a) Absolute resistance vs. strain for the non-sonicated ETC samples before activation. (b) Photographs of non-sonicated and sonicated ETC samples after 240% strain and subsequent release. (c) Absolute resistance vs. strain for the non-sonicated sample (after activation) and sonicated simple. (d) Resistance evolution over multiple 240% strain cycles.

In the tensile test (Figure S8a), the non-sonicated sample initially exhibited an extremely high resistance (>20 MΩ). As strain increased beyond 80%, the resistance dropped significantly to approximately 2 Ω. This suggests that stretching mechanically activated the conductive network by breaking the oxide layers on EGaIn particles, forming new conductive pathways. Nevertheless, this activation process was accompanied by EGaIn leakage (Figure S8b). By contrast, the sonicated samples exhibited a significantly reduced particle size, forming a more stable conductive network. Even after recovering from 460% strain, no leakage was observed, demonstrating better reliability.

We also conducted resistance-strain tests on both the mechanically activated non-sonicated samples and the sonicated samples (Figure S8c). The results indicate that after initial activation, the non-sonicated samples exhibited a resistance-strain trend similar to that of the sonicated samples. However, due to the leakage of EGaIn, the non-sonicated samples consistently showed higher resistance than the sonicated samples.

Besides, we investigated the resistance changes of both sonicated and non-sonicated samples (after activation) during 0–240% cyclic tensile tests (Figure S8d). The resistance of both the sonicated and non-sonicated samples remained constant throughout strain cycles. To be noted, the non-sonicated samples showed higher resistance than the sonicated ones.


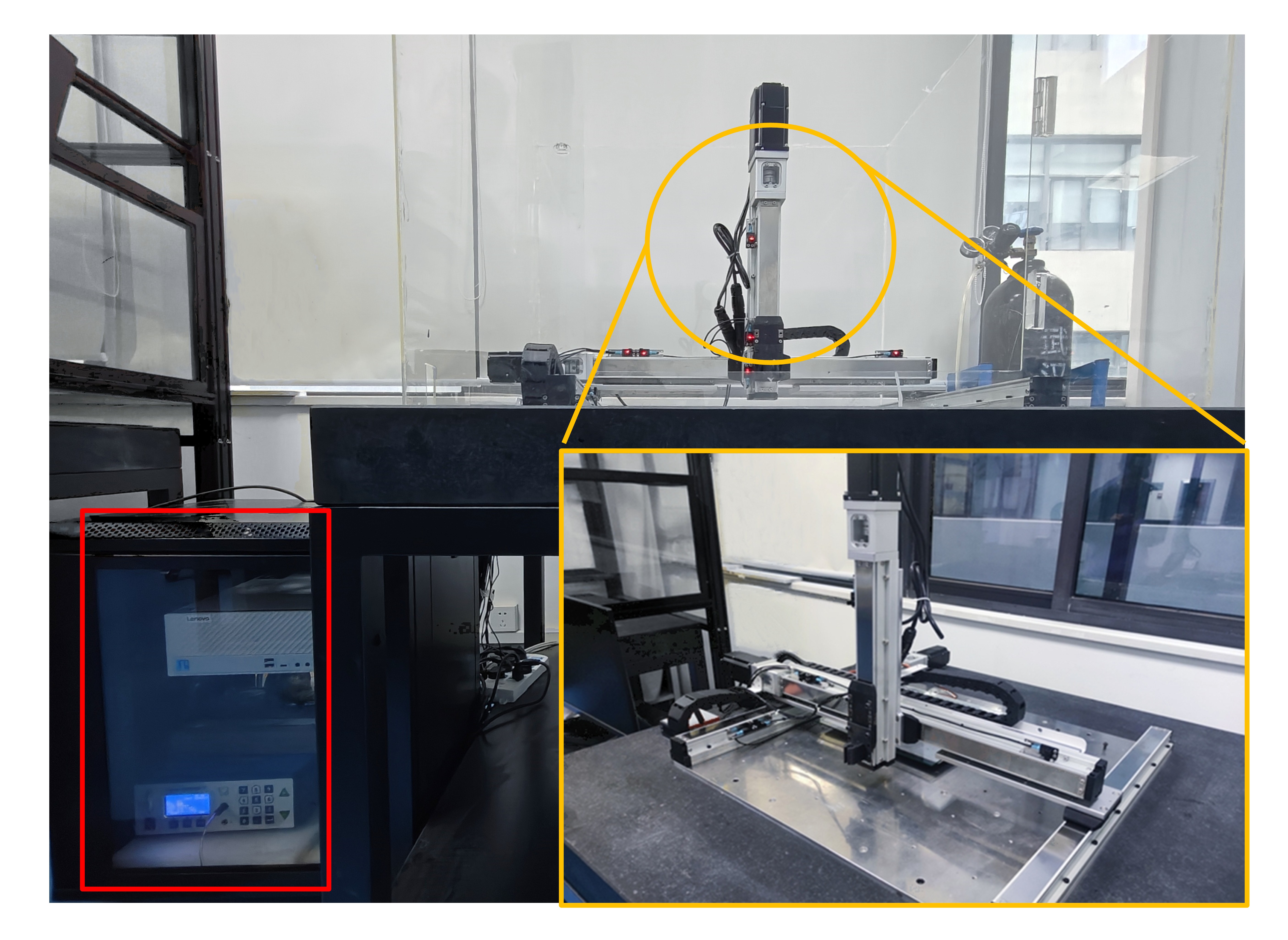


**Figure S9** The photograph of Direct Ink Writing (DIW) system composed by three-axis motion platform (in orange wireframe), motion controller and pneumatic liquid dispenser (in red wireframe).


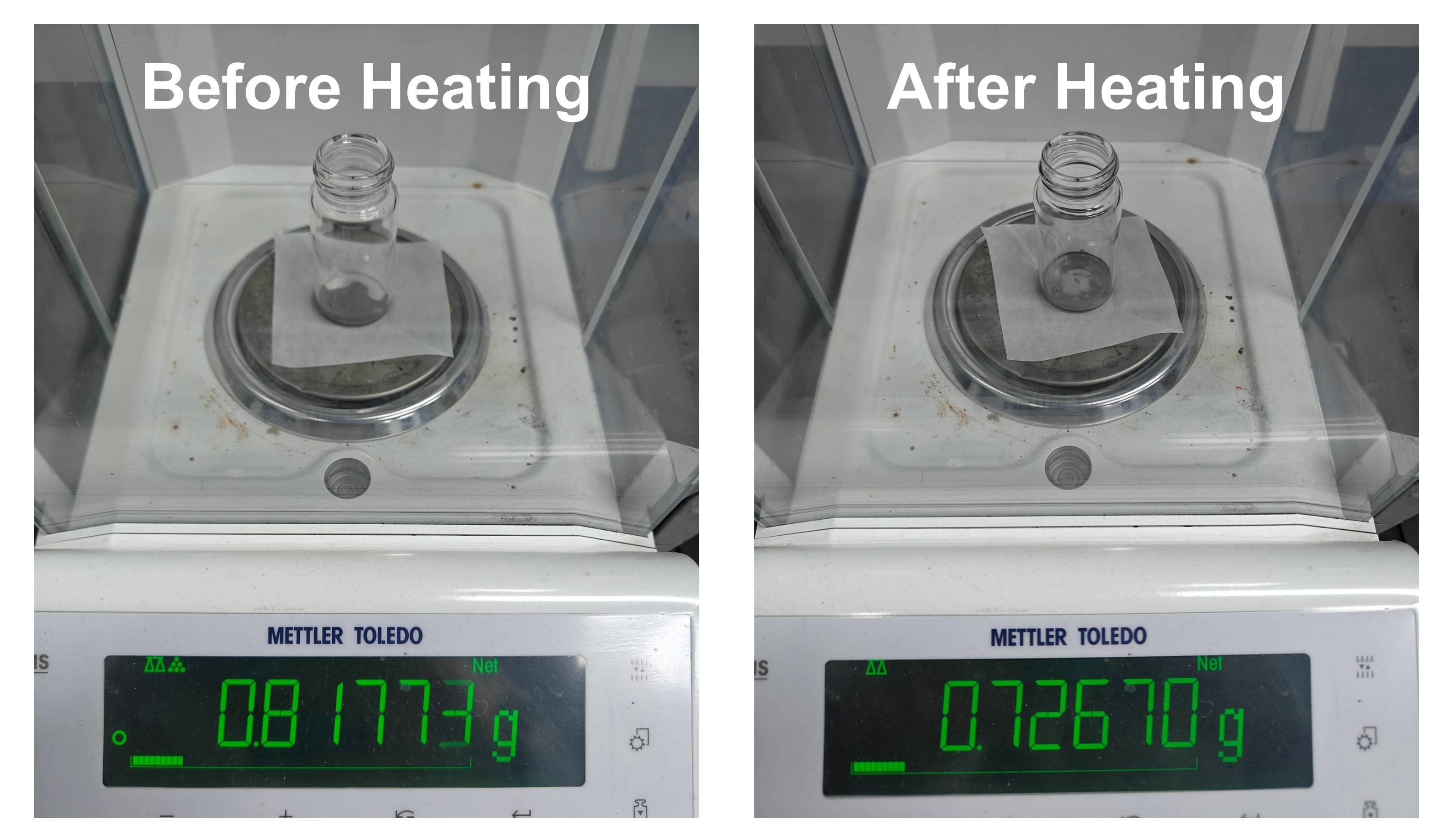


Figure S10. The thermogravimetric analysis of ETC

The ink formulation was designed with DMAC comprising 11 wt% of the total mass (40.7% of the total volume). Before heating, the viscosity of the ink was measured to be 153 Pa·s (Figure 1b in the main text). To verify the complete removal of DMAC, we conducted a thermogravimetric analysis (TGA). TGA results showed a mass reduction from 0.818 g to 0.727 g after heating at 60°C for 4 hours, matching the initial 11.0 wt% DMAC content in the ink formulation. This demonstrates that the entire 11 wt% DMAC has completely evaporated, leaving the composite in a dry state. Consequently, the viscosity of the ink after heating was not measured, as the composite is no longer in a liquid state.


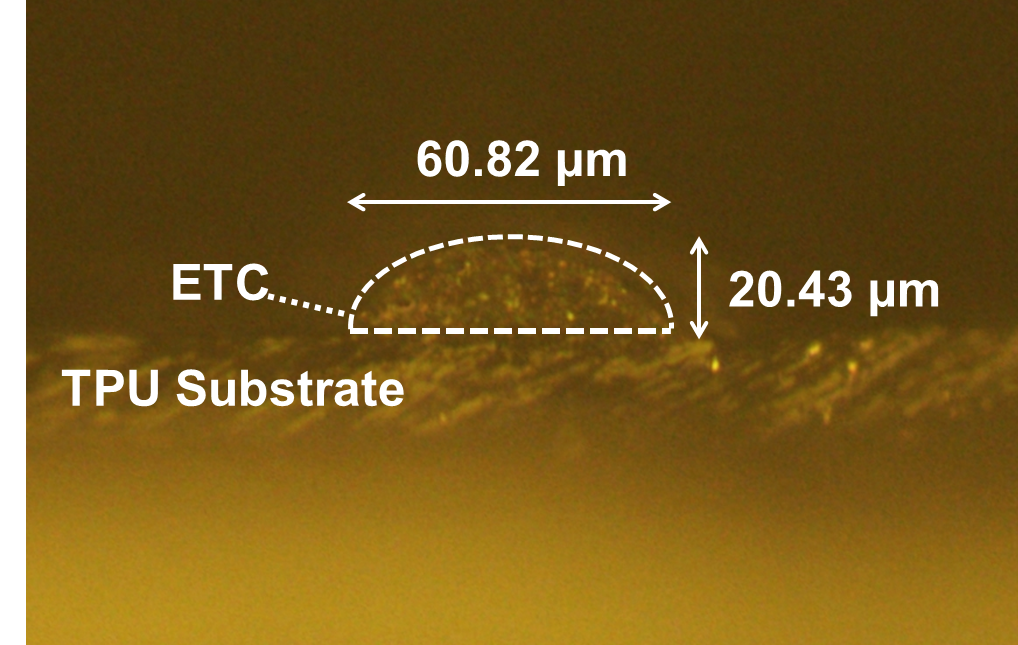


**Figure S11** The cross-section microscopy image of the ETC line printed by nozzle with 60 μm inner diameter.


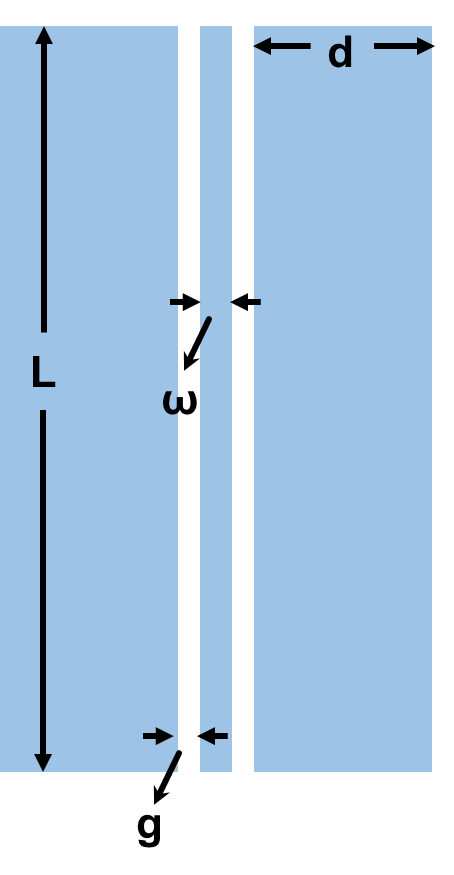


**Figure S12** The schematic diagram of the coplanar waveguide structure. (L=25mm, g=ω=0.2mm, d=5mm.)

For the electromagnetic test, the two coplanar-waveguide samples were prepared on a rigid glass substrate by DIW printing and laser engraving, respectively.

Then, a vector network analyzer (VNA) was used to test their S_21_ parameters and calculate the attenuation constant by equation 5 in the main text.


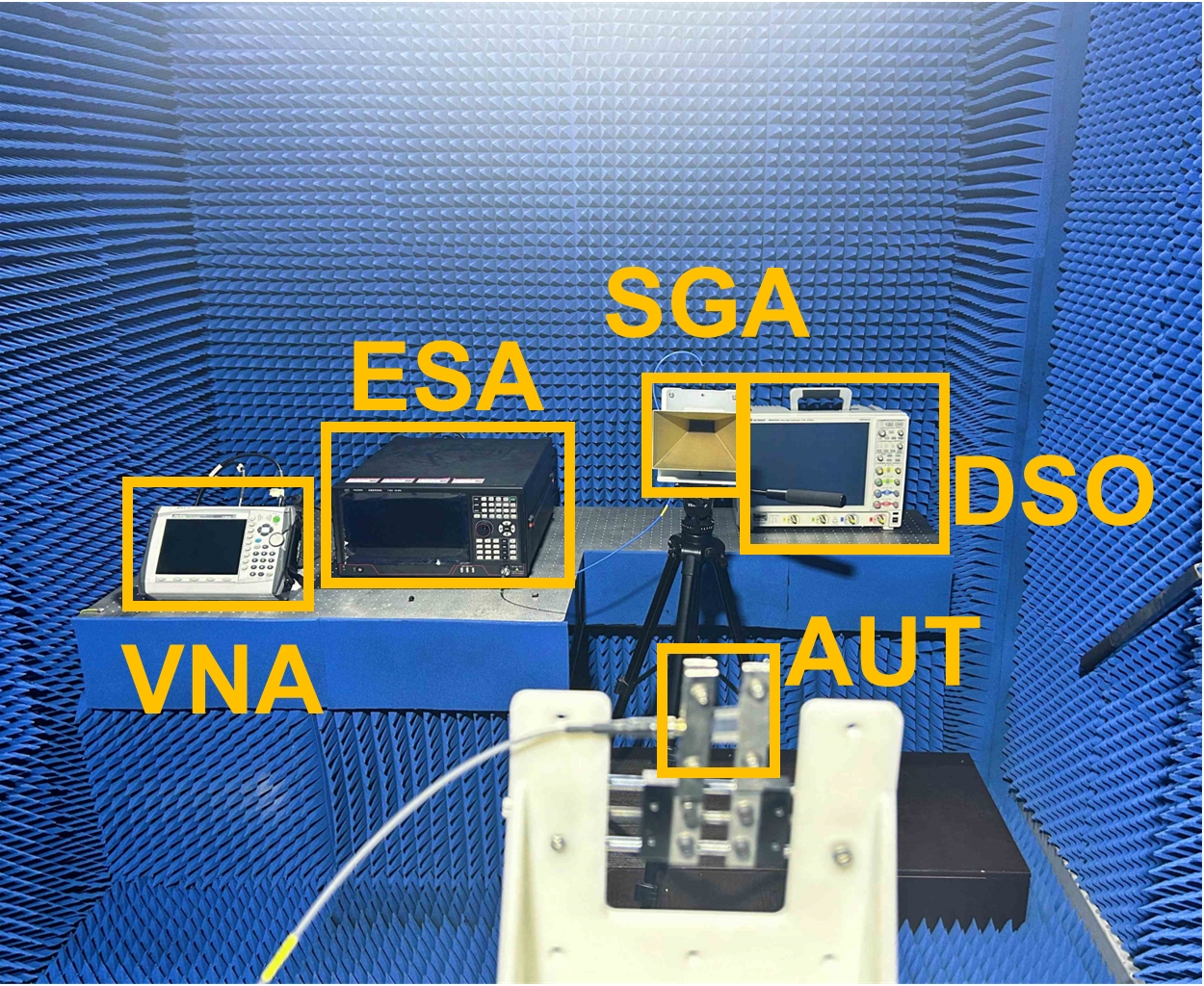


**Figure S13** The photograph of the experimental set-up in an anechoic chamber. AUT: antenna under test; VNA: vector network analyzer; ESA: electrical signal analyzer; SGA: standard-gain antenna; DSO: digital storage oscilloscope.

To minimize the electromagnetic wave scattering from the test environment, the RF performances of the printed antenna including operating bandwidth, radiation pattern, peak gain and radiation efficiency are all measured in the microwave anechoic chamber at Huazhong University of Science and Technology.


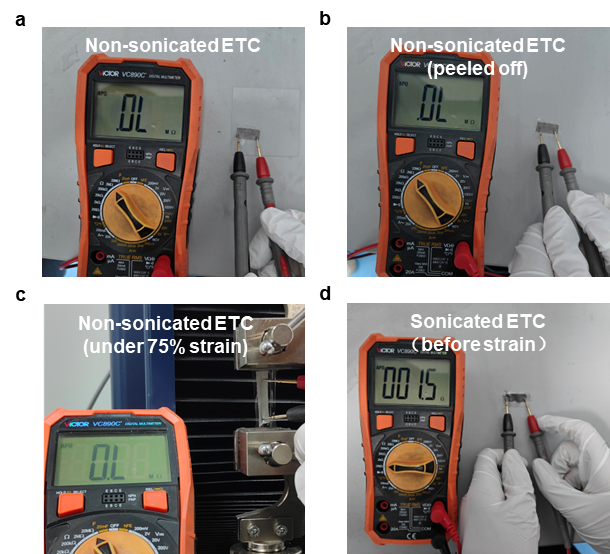


**Figure S14** Photograph of ETC’s resistance measurements using a multimeter: (a) Non-sonicated ETC on TPU substrate attached to the glass, (b) Non-sonicated ETC peeled off from the glass, (c) Non-sonicated ETC under 75% strain, and (d) Sonicated ETC before strain.

We have illustrated the samples under different treatment conditions including the printing, peeling off, 75% stretching and ultrasonic activation of the proposed composite (Figure S14). To be noted, the composite material exhibits conductivity only after ultrasonic activation. (“0L” on the screen of the multimeter means > 20 MΩ) This result indicates that simple peeling or stretching to a certain extent is insufficient for activating the EGaIn-TPU composite. The ultrasonic-induced reorganization process directly facilitates activation and conductivity.


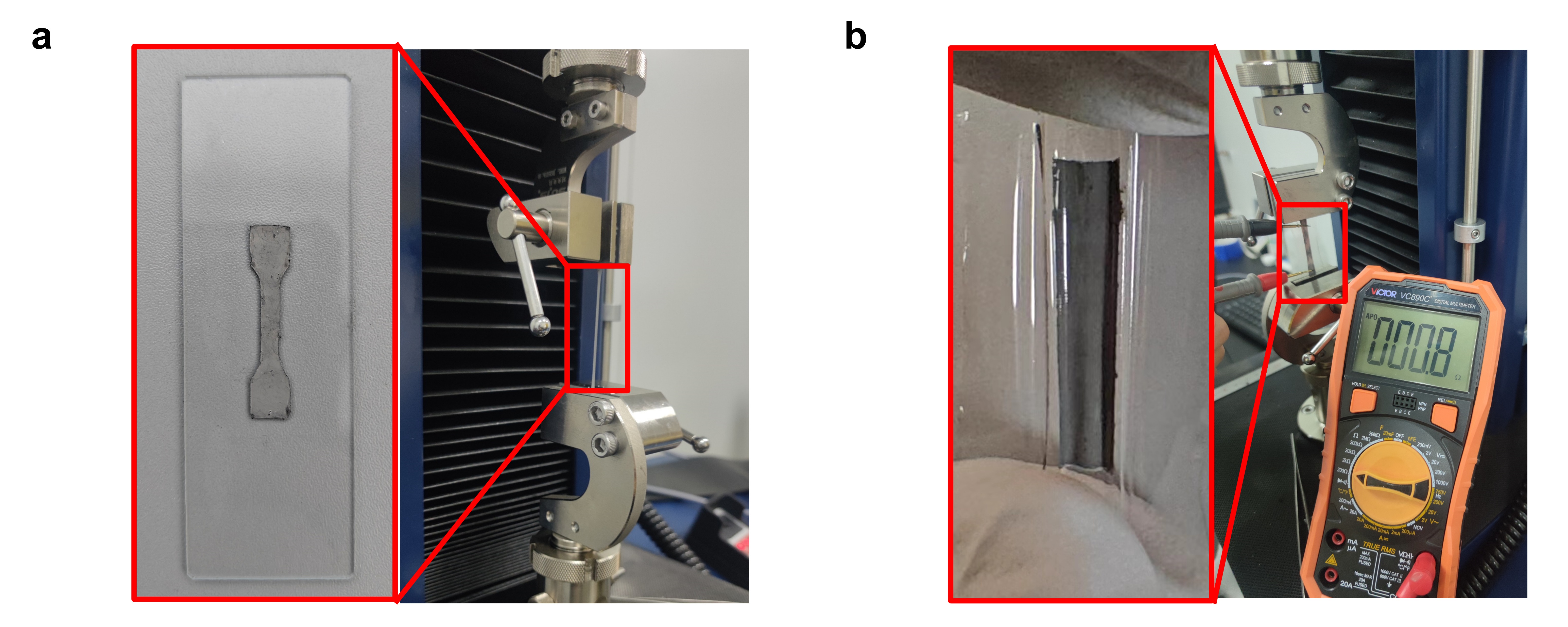


**Figure S15** The ETC printed as a rectangular resistor for the tensile test.

Figure S15 shows a printed resistor under strain, and its resistance is measured by a multimeter to evaluate the resistance of the ETC during the stretching process. Test samples were rectangular patterns (8 mm×20 mm) printed on a TPU substrate. Resistance changes were recorded in real-time using a multimeter during tensile testing on the universal testing machine.

**Reference**

[1] L. Teng, S. Ye, S. Handschuh‐Wang, X. Zhou, T. Gan, X. Zhou, *Advanced Functional Materials* **2019**, 29, 1808739.

[2] J. Tang, X. Zhao, J. Li, R. Guo, Y. Zhou, J. Liu, *ACS applied materials & interfaces* **2017**, 9, 35977.

[3] B. Ma, C. Xu, J. Chi, J. Chen, C. Zhao, H. Liu, *Advanced Functional Materials* **2019**, 29, 1901370.

[4] J.-E. Park, H. S. Kang, J. Baek, T. H. Park, S. Oh, H. Lee, M. Koo, C. Park, *ACS Nano* **2019**, 13, 9122.

[5] C. J. Thrasher, Z. J. Farrell, N. J. Morris, C. L. Willey, C. E. Tabor, *Advanced Materials* **2019**, 31, 1903864.

[6] S. H. Kim, A. Basir, R. Avila, J. Lim, S. W. Hong, G. Choe, J. H. Shin, J. H. Hwang, S. Y. Park, J. Joo, C. Lee, J. Choi, B. Lee, K.-S. Choi, S. Jung, T.-i. Kim, H. Yoo, Y. H. Jung, *Nature* **2024**, 629, 1047.

[7] Z. Chen, J. Xi, W. Huang, M. M. F. Yuen, *Scientific Reports* **2017**, 7, 10958.

[8] J. Zhu, S. Zhang, N. Yi, C. Song, D. Qiu, Z. Hu, B. Li, C. Xing, H. Yang, Q. Wang, H. Cheng, *Nano-Micro Letters* **2021**, 13, 108.

[9] A. M. Hussain, F. A. Ghaffar, S. I. Park, J. A. Rogers, A. Shamim, M. M. Hussain, *Advanced Functional Materials* **2015**, 25, 6565.

[10] F. Liu, Y. Chen, H. Song, F. Zhang, Z. Fan, Y. Liu, X. Feng, J. A. Rogers, Y. Huang, Y. Zhang, *Small* **2019**, 15, e1804055.

[11] B. S. Kim, K. Y. Shin, J. B. Pyo, J. Lee, J. G. Son, S. S. Lee, J. H. Park, *ACS Appl Mater Interfaces* **2016**, 8, 2582.

[12] L. Song, A. C. Myers, J. J. Adams, Y. Zhu, *ACS Applied Materials & Interfaces* **2014**, 6, 4248.

[13] A. Lamminen, K. Arapov, G. de With, S. Haque, H. G. O. Sandberg, H. Friedrich, V. Ermolov, *IEEE Antennas and Wireless Propagation Letters* **2017**, 16, 1883.

[14] C. Shi, W. Zhigang, P. Hallbjorner, K. Hjort, A. Rydberg, *IEEE Transactions on Antennas and Propagation* **2009**, 57, 3765.

[15] G. J. Hayes, J. H. So, A. Qusba, M. D. Dickey, G. Lazzi, *IEEE Transactions on Antennas and Propagation* **2012**, 60, 2151.

[16] M. R. Ramli, S. Ibrahim, Z. Ahmad, I. S. Z. Abidin, M. F. Ain, *ACS Applied Materials & Interfaces* **2019**, 11, 28033.

[17] M. Li, Y. Tao, P. Yu, *Applied Sciences*, 10.3390/app13084950

[18] Q. Yi, S. Gao, A. Sambell, *IEEE Transactions on Microwave Theory and Techniques* **2006**, 54, 2723.

[19] L. Zhu, N. Liu, *Electromagnetic Science* **2023**, 1, 1.
